# CuCl-catalyzed aerobic oxidation of 2,3-allenols to 1,2-allenic ketones with 1:1 combination of phenanthroline and bipyridine as ligands

**DOI:** 10.3762/bjoc.7.51

**Published:** 2011-04-07

**Authors:** Shuxu Gao, Yu Liu, Shengming Ma

**Affiliations:** 1 Shanghai Key Laboratory of Green Chemistry and Chemical Processes, Department of Chemistry, East China Normal University, 3663 North Zhongshan Road, Shanghai 200062, P. R. China; 2 State Key Laboratory of Organometallic Chemistry, Shanghai Institute of Organic Chemistry, Chinese Academy of Sciences, 345 Lingling Lu, Shanghai 200032, P. R. China. Fax: (+86)-21-6260-9305

**Keywords:** allenic ketone, allenol, Cu(I) catalyst, oxidation

## Abstract

A protocol has been developed to prepare 1,2-allenyl ketones using molecular oxygen in air or pure oxygen as the oxidant from 2,3-allenylic alcohols with moderate to good yields under mild conditions. In this reaction CuCl (20 mol %) with 1,10-phenanthroline (10 mol %) and bipyridine (10 mol %) was used as the catalyst. It is interesting to observe that the use of the mixed ligands is important for the higher yields of this transformation: With the monoligand approach developed by Markó et al., the yields are relatively lower.

## Introduction

The oxidation of alcohols is one of many fundamental reactions in organic synthesis [1–2]. Usually, stoichiometric oxidants such as MnO_2_ [3], PCC [4], PDC [4], etc. were employed for this type of transformation. However, the cost and the byproducts derived from these reagents cause economic and environmental problems [5]. In the past decades, much attention has been paid to catalytic oxidation of alcohols using molecular oxygen as the oxidant with Pd [6–10], Cu [11–13], Ru [14–15] as the catalysts.

1,2-Allenic ketones have become very useful in organic synthesis [16–33]. Current methods for the oxidation of allenic alcohols to ketones include oxidation with MnO_2_ [30,34–35], Swern oxidation [17,24] or Dess–Martin oxidation [16–17,24–25,28,31–33]: Catalytic aerobic oxidation has not so far been reported. Due to the synthetic potential of 1,2-allenyl ketones, it is desirable to develop an aerobic oxidation protocol for 2,3-allenols. In this paper we wish to report the CuCl-catalyzed aerobic oxidation of 2,3-allenols by applying a mixed ligand approach using copper as the catalyst [12–13].

## Results and Discussion

After screening the Pd- [6–10] and Ru-catalyzed [14–15] protocols without success, we began a study of the oxidation of 2-hexyl-1-phenylbuta-2,3-diene-1-ol (**1a**) with O_2_ based on the pioneering study of oxidation of normal simple alcohols by Markó et al. [12]. Under the original conditions [12], the expected allenylic ketone **2a** was obtained in 59% yield when CuCl and 1,10-phenanthroline were used (Table 1, entry 1). A series of bases and solvents were then screened for the oxidation of **1a**. The results are summarized in Table 1 and Table 2. We found that (1) K_2_CO_3_ is the most effective base (Table 1, entry 1) and that organic bases such as NEt_3_ and DBU are generally ineffective (Table 1, entries 6 and 7); (2) toluene is the best solvent (Table 2).

**Table 1:** Screening of bases for the CuCl-catalyzed oxidation of **1a**^a^.

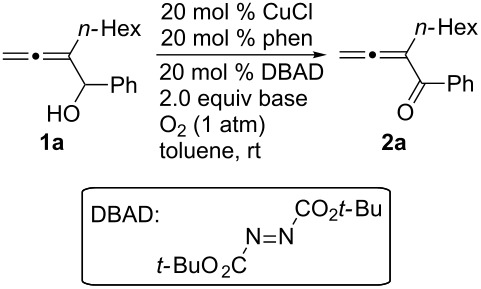			

entry	base	time (h)	yield of **2a** (%)^b^

1	K_2_CO_3_	40	59^c,d^
2	Na_2_CO_3_	48	22^e^
3	Cs_2_CO_3_	35	27^d,f,g^
4	KHCO_3_	45	15^h^
5	KOH^i^	48	39^j^
6	NEt_3_	45	NR^k^
7	DBU	45	NR^l^

^a^The reaction was carried out using 0.3 mmol of **1a**, 20 mol % of CuCl, 20 mol % of phen, 20 mol % of DBAD, and 2.0 equiv of base in 3 mL of toluene under 1 atm of oxygen unless otherwise stated. ^b1^H NMR yield using CH_2_Br_2_ as the internal standard. ^c^1.0 equiv K_2_CO_3_ was used. ^d^Isolated yield. ^e^50% of **1a** was recovered as determined by ^1^H NMR analysis. ^f^15 mol % of catalyst was used. ^g^32% of **1a** was recovered by column chromatography. ^h^53% of **1a** was recovered as determined by ^1^H NMR analysis. ^i^100 mg of 3 Å MS and 20 mol % of KOH was used. ^j^28% of **1a** was recovered as determined by ^1^H NMR analysis. ^k^70% of **1a** was recovered as determined by ^1^H NMR analysis. ^l^72% of **1a** was recovered by column chromatography.

**Table 2:** Screening of solvents for the CuCl-catalyzed oxidation of **1a**^a^.

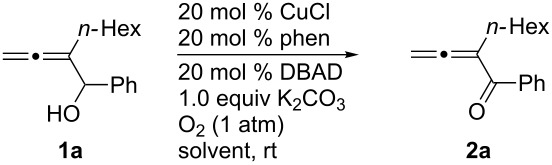			

entry	solvent	time (h)	yield of **2a** (%)

1	toluene	40	59^b^
2	CH_3_CN	48	NR^c^
3	DCE	47	31^d^
4	CHCl_3_	47	20^e^
5	DMF	47	NR^f^

^a^The reaction was carried out using 0.3 mmol of **1a**, 20 mol % of CuCl, 20 mol % of phen, 20 mol % of DBAD, and 1.0 equiv of K_2_CO_3_ in 3 mL of solvent under 1 atm of oxygen. ^b^Isolated yield. ^c^64% of **1a** was recovered as determined by ^1^H NMR analysis. ^d1^H NMR yield using CH_2_Br_2_ as the internal standard. ^e^78% of **1a** was recovered as determined by ^1^H NMR analysis. ^f^76% of **1a** was recovered as determined by ^1^H NMR analysis.

In order to improve the yield further, we examined the effect of ligands. When 2,2'-bipyridine, which has a weaker coordinating ability, was used [36], the yield of **2a** was lower (Table 3, entry 2). With 4,7-diphenyl-1,10-phenanthroline the yield was slightly improved to 66% (Table 3, entry 3). These experimental results obviously indicated that the CuCl-catalyzed oxidation of allenic alcohol was influenced by the coordinating ability of nitrogen ligands. Consequently, we carried out the reaction with a mixture of a stronger coordinating ligand together with a relatively weaker coordinating ligand. Indeed, it was interesting to observe that when 4,7-diphenyl-1,10-phenanthroline and 2,2'-bipyridine were mixed in the ratio of 1:1 [37–38], the isolated yield was improved to 82% (Table 3, entry 6). The yield with 1,10-phenanthroline and 2,2'-bipyridine (1:1) was 83% (Table 3, entry 10). However, 4,7-diphenyl-1,10-phenanthroline is relatively expensive (1 g, $ 94, Aldrich), so the cheaper 1,10-phenanthroline (5 g, $ 26.4, Aldrich) was used for further study. The effect of ratio of 1,10-phenanthroline vs 2,2'-bipyridine on the yield was also studied: A ratio of 1:1 proved to be the best (Table 3, entries 7–12). This may be explained by considering that the coordination of 2,2'-bipyridine is important for the formation of the catalytically active species and may be easily replaced with that of the alcohol. We also tried *N*-methylimidazole (NMI), which was used in oxidation of primary aliphatic alcohols reported by Markó et al. [13], however, both the turnover and yield were low (Table 3, entry 14). Both 2,9-dimethyl-4,7-diphenyl-1,10-phenanthroline and iPr-Pybox were ineffective in this reaction (Table 3, entries 4 and 5).

**Table 3:** Screening for different nitrogen ligands in the CuCl-catalyzed oxidation of **1a**^a^.

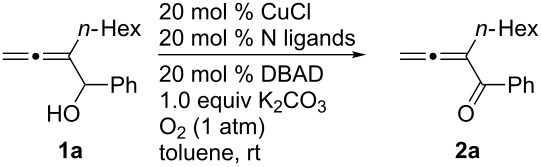				

entry	ligand 1 (mol %)	ligand 2 (mol %)	time (h)	yield of **2a** (%)^b^

1	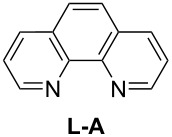	—	40	61
2	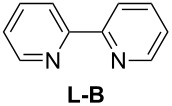	—	46	43^c^
3	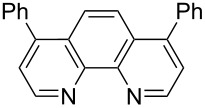	—	14.5	66
4	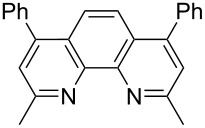	—	45	NR^d^
5	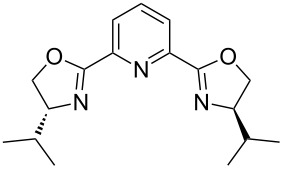	—	24	NR^e^
6	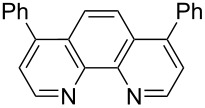 (10)	**L-B** (10)	45.5	82
7	**L-A** (17.5)	**L-B** (2.5)	40	79
8	**L-A** (15.0)	**L-B** (5.0)	40	65
9	**L-A** (12.5)	**L-B** (7.5)	40	82
10	**L-A** (10.0)	**L-B** (10.0)	42	83
11	**L-A** (7.5)	**L-B** (12.5)	42	78
12	**L-A** (5.0)	**L-B** (15.0)	40	73
13	**L-A** (2.5)	**L-B** (17.5)	40	68
14^f^	**L-A** (5.0)	**NMI** (7.0)	35	17

^a^The reaction was carried out using 0.3 mmol of **1a**, 20 mol % of CuCl, 20 mol % of nitrogen ligand, 20 mol % of DBAD, and 1.0 equiv of K_2_CO_3_ in 3 mL of toluene under 1 atm of oxygen. ^b1^H NMR yields determined by 300 MHz, ^1^H NMR analysis using CH_2_Br_2_ as the internal standard. ^c^52% of **1a** was recovered as determined by ^1^H NMR analysis. ^d^87% of **1a** was recovered as determined by ^1^H NMR analysis. ^e^82% of **1a** was recovered as determined by ^1^H NMR analysis. ^f^The reaction was carried out using 0.5 mmol of **1a**, 5 mol % of CuCl, 5 mol % of *t*-BuOK, 5 mol % of DBAD and the indicated ligands in 5 mL of C_6_H_5_F at 70 °C under 1 atm of oxygen. 52% of **1a** was recovered as determined by ^1^H NMR analysis.

Some other Cu(I) catalysts, such as CuBr, CuI, and CuCN were also investigated, but no higher yield was achieved (Table 4). Further studies led to the observation that air (300 psi, 35 °C (oil bath)) could be used instead of pure oxygen (1 atm, 15–24 °C) to shorten the reaction time from 40 to 10 hours and the yield was similar (86%) (Table 5, entry 1). Thus, 20 mol % of CuCl, 10 mol % of 1,10-phenanthroline, 10 mol % of 2,2'-bipyridine and 50 mol % of K_2_CO_3_ in toluene with air (300 psi, 35 °C) as the oxidant were defined as the standard conditions.

**Table 4:** Screening of other Cu(I) sources.

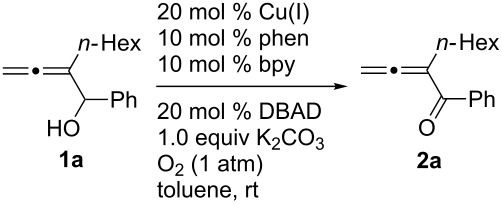			

entry	Cu(I)	time (h)	isolated yield of **2a**

1	CuBr	40	68%
2	CuI	40	49%
3	CuCN	41	45%

**Table 5:** The CuCl-catalyzed oxidation of allenic alcohols using air as the oxidant^a^.

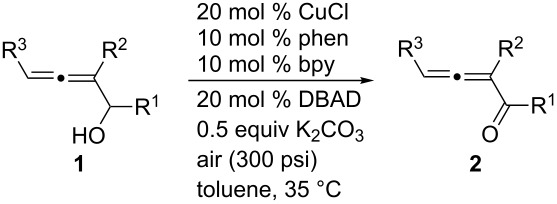					

entry	substrate			time (h)	yield (%)^b^
	R^1^	R^2^	R^3^		

1	Ph	*n-*C_6_H_13_	H (**1a**)	10	86 **(2a)**
2	*p-*EtC_6_H_4_	*n-*Pr	H (**1b**)	10	83 **(2b)**
3	*p-*BrC_6_H_4_	*n-*Pr	H (**1c**)	6	80 **(2c)**
4	*p-*ClC_6_H_4_	*n-*C_6_H_13_	H (**1d**)	6	78 **(2d)**
5	*p-*O_2_NC_6_H_4_	*n-*C_6_H_13_	H (**1e**)	6	63 **(2e)**
6	3-furanyl	*n-*C_6_H_13_	H (**1f**)	11	61 **(2f)**
7	3-thienyl	*n-*C_5_H_11_	H (**1g**)	8.5	73 **(2g)**
8	1-naphthyl	Me	H (**1h**)	11	74 **(2h)**
9	Ph	allyl	H (**1i**)	11	75 **(2i)**
10	Ph	Bu	*n*-C_5_H_11_ (**1j**)	11	91 **(2j)**

^a^The reaction was carried out using 0.3 mmol of **1**, 20 mol % of CuCl, 10 mol % of phen, 10 mol % of bpy, 20 mol % of DBAD, and 0.5 equiv of K_2_CO_3_ in 3 mL of toluene, air (300 psi, 35 °C (oil bath)). ^b^Isolated yields.

Under the standard conditions a series of 1-aryl-2,3-allenols were oxidized to the corresponding 1,2-allenic aryl ketones: A *para*-nitro group led to a 63% yield of **2e** (Table 5, entry 5); heteroaryl groups such as furanyl and thienyl were also tolerated under the reaction conditions, affording the corresponding allenic ketones **2f** and **2g** in 61% and 73% yields, respectively (Table 5, entries 6 and 7), whilst the reaction of 1-naphthyl-substituted **1h** afforded **2h** in 74% yield (Table 5, entry 8). Tri-substituted allenic alcohol **1j** was also oxidized to the corresponding allenic ketone **2j** in 91% yield (Table 5, entry 10).

**Scheme 1:**
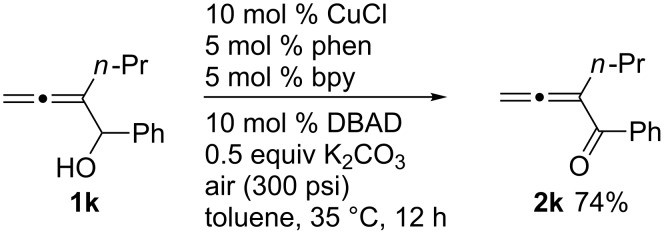
1 gram scale reaction of allenol **1k**.

The reaction may be easily carried out on a 1 g scale: the oxidation of allenol **1k** afforded the corresponding allenic ketone **2k** in 74% yield in 12 hours with just 10 mol % of CuCl and 5 mol % each of 1,10-phenanthroline and 2,2'-bipyridine (Scheme 1).

**Scheme 2:**
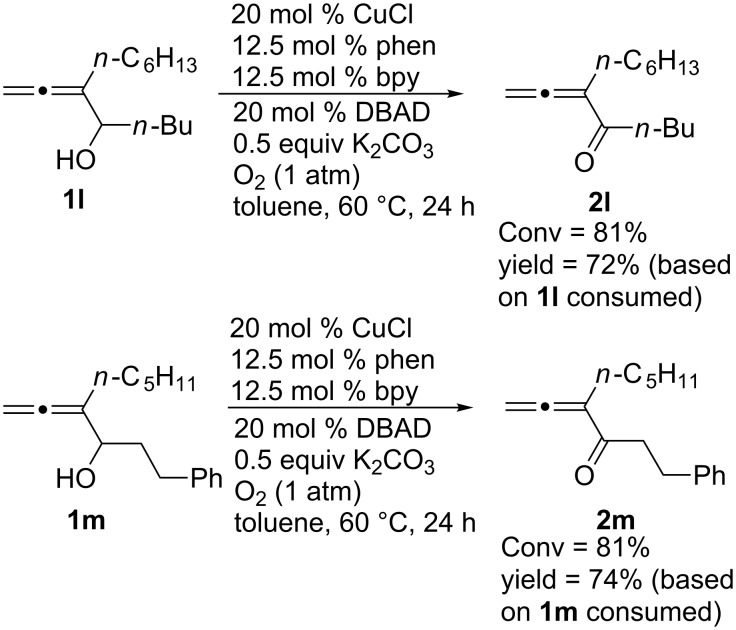
The oxidation of **1l** and **1m** under 1 atm of oxygen.

When the reaction of 1-alkyl-substituted-2,3-allenols oxidation was conducted under 1 atm of oxygen at 60 °C, 81% of conversion was observed and the corresponding allenic ketones **2l** and **2m** were obtained in 58% and 60% isolated yields (72% and 74% based on the starting material consumed), respectively (Scheme 2). As a comparison, it should be noted that when **1l** was oxidized with air (300 psi, 60 °C), the allenic ketone **2l** was formed in 43% ^1^H NMR yield with 73% conversion of **1l** within 10 hours.

## Conclusion

In conclusion, we have developed a method for the aerobic oxidation of 2,3-allenols, which uses molecular oxygen in air or pure oxygen as the oxidant. In this reaction, CuCl with a 1:1 ratio of 1,10-phenanthroline and bipyridine was used as the catalyst to provide the best results. A series of 1,2-allenic ketones were obtained in moderate to good yields under mild conditions. Compared to the traditional monoligand approach, allenols are obviously unique demanding a mixed ligands approach for better yields probably as a consequence of the coordinating ability of the allene moiety. Further study in this area is being pursued in this laboratory.

## Experimental

### General experimental methods for starting materials

The starting allenols **1a–e**, **1i**, **1k**, **1l**, **1m** were prepared via the reaction of propargyl bromides and corresponding aldehydes in the presence of SnCl_2_ and NaI in DMF [39–40]; allenols **1f** [41], **1g** [42], **1h** [43], and **1j** [44] were prepared as reported. These starting allenols were purified by flash chromatography before use.

### General experimental procedure for the aerobic oxidation of allenic alcohols

#### 2-Hexyl-1-phenylbuta-2,3-dien-1-one (**2a**)

Typical procedure: 1,10-phenanthroline (5.5 mg, 0.03 mmol), 2,2'-bipyridine (4.7 mg, 0.03 mmol), CuCl (5.9 mg, 0.06 mmol), K_2_CO_3_ (20.6 mg, 0.15 mmol), and 1.5 mL of dry toluene were added successively into an oven dried reaction vessel (sealed with a stopper to isolate the contents from atmospheric moisture). The resulting mixture was stirred at rt for 0.5 h. Then the stopper was removed to add DBAD (13.7 mg, 0.06 mmol), 2-hexyl-1-phenylbuta-2,3-dien-1-ol (69.6 mg, 0.3 mmol), and 1.5 mL of dry toluene. The reaction vessel was then transferred to an autoclave, which was charged with air to a pressure of 300 psi, and stirred at 35 °C (oil bath). After 10 h, the pressure was carefully released in the hood, the mixture filtered through a short column of silica gel (100–140 mesh) and washed with diethyl ether. Evaporation of the solvent and flash chromatography on silica gel (eluent: petroleum ether/diethyl ether = 30:1) afforded **2a** (59.3 mg, 86%): liquid; ^1^H NMR (300 MHz , CDCl_3_) δ 7.76 (d, *J* = 7.2 Hz, 2H), 7.49 (t, *J* = 7.4 Hz, 1H), 7.39 (t, *J* = 7.2 Hz, 2H), 5.04 (t, *J* = 2.7 Hz, 2H), 2.46–2.35 (m, 2H), 1.58–1.45 (m, 2H), 1.45–1.20 (m, 6H), 0.89 (t, *J* = 6.6 Hz, 3H) ppm; ^13^C NMR (75.4 MHz, CDCl_3_) δ 217.0, 194.9, 138.3, 131.9, 129.0, 127.8, 106.9, 79.3, 31.6, 28.9, 27.8, 22.6, 14.0 ppm; MS (*m*/*z*) 228 (M^+^, 7.25), 105 (100); IR (neat) 2927, 2857, 1933, 1653, 1599, 1450, 1312, 1273 cm^−1^; HRMS-EI (*m*/*z*) calcd for C_16_H_20_O^+^ [M^+^]: 228.1514; found: 228.1517.

#### 2-Propyl-1-(4-ethylphenyl)buta-2,3-dien-1-one (**2b**)

The reaction of 1,10-phenanthroline (5.3 mg, 0.03 mmol), 2,2'-bipyridine (4.6 mg, 0.03 mmol), CuCl (5.9 mg, 0.06 mmol), K_2_CO_3_ (20.8 mg, 0.15 mmol), dry toluene (1.5 mL), DBAD (13.7 mg, 0.06 mmol), and 2-propyl-1-(4-ethylphenyl)buta-2,3-dien-1-ol (64.7 mg, 0.3 mmol)/dry toluene (1.5 mL) afforded **2b** (53.0 mg, 83%): liquid; ^1^H NMR (300 MHz, CDCl_3_) δ 7.73 (d, *J* = 8.1 Hz, 2H), 7.22 (d, *J* = 8.1 Hz, 2H), 5.05 (t, *J* = 2.9 Hz, 2H), 2.69 (q, *J* = 7.6 Hz, 2H), 2.44–2.32 (m, 2H), 1.63–1.45 (m, 2H), 1.25 (t, *J* = 7.5 Hz, 3H), 0.99 (t, *J* = 7.4 Hz, 3H) ppm; ^13^C NMR (75.4 MHz, CDCl_3_) δ 216.5, 194.4, 148.8, 135.8, 129.3, 127.3, 106.5, 79.1, 30.1, 28.8, 21.1, 15.1, 13.7 ppm; MS (*m*/*z*) 214 (M^+^, 1.75), 133 (100); IR (neat) 2964, 2931, 2872, 1933, 1650, 1607, 1458, 1414, 1273, 1182, 1058 cm^−1^; HRMS-EI (*m*/*z*) calcd for C_15_H_18_O^+^ [M^+^]: 214.1358; found: 214.1360.

#### 2-Propyl-1-(4-bromophenyl)buta-2,3-dien-1-one (**2c**)

The reaction of 1,10-phenanthroline (5.4 mg, 0.03 mmol), 2,2'-bipyridine (4.6 mg, 0.03 mmol), CuCl (5.9 mg, 0.06 mmol), K_2_CO_3_ (20.6 mg, 0.15 mmol), dry toluene (1.5 mL), DBAD (13.8 mg, 0.06 mmol), and 2-propyl-1-(4-bromophenyl)buta-2,3-dien-1-ol (80.3 mg, 0.3 mmol)/dry toluene (1.5 mL) afforded **2c** (64.1 mg, 80%): liquid; ^1^H NMR (300 MHz, CDCl_3_) δ 7.62 (d, *J* = 8.7 Hz, 2H), 7.52 (d, *J* = 8.4 Hz, 2H), 5.07 (t, *J* = 2.9 Hz, 2H), 2.41–2.30 (m, 2H), 1.60–1.45 (m, 2H), 0.97 (t, *J* = 7.5 Hz, 3H) ppm; ^13^C NMR (75.4 MHz, CDCl_3_) δ 217.0, 193.7, 137.0, 131.1, 130.5, 126.7, 106.7, 79.7, 29.7, 21.1, 13.7 ppm; MS (*m*/*z*) 266 (M^+^ (^81^Br), 1.68), 264 (M^+^ (^79^Br), 1.76), 185 (100); IR (neat) 2961, 2929, 2870, 1931, 1653, 1585, 1458, 1391, 1270, 1071, 1010 cm^−1^; HRMS-EI (*m*/*z*) calcd for C_13_H_13_O^81^Br^+^ [M^+^]: 266.0129; found: 266.0136.

#### 2-Hexyl-1-(4-chlorophenyl)buta-2,3-dien-1-one (**2d**)

The reaction of 1,10-phenanthroline (5.5 mg, 0.03 mmol), 2,2'-bipyridine (4.8 mg, 0.03 mmol), CuCl (6.0 mg, 0.06 mmol), K_2_CO_3_ (20.9 mg, 0.15 mmol), dry toluene (1.5 mL), DBAD (13.8 mg, 0.06 mmol), and 2-hexyl-1-(4-chlorophenyl)buta-2,3-dien-1-ol (79.6 mg, 0.3 mmol)/dry toluene (1.5 mL) afforded **2d** (62.0 mg, 78%): liquid; ^1^H NMR (300 MHz, CDCl_3_) δ 7.70 (d, *J* = 8.4 Hz, 2H), 7.35 (d, *J* = 8.4 Hz, 2H), 5.06 (t, *J* = 2.6 Hz, 2H), 2.43–2.32 (m, 2H), 1.55–1.42 (m, 2H), 1.43–1.18 (m, 6H), 0.88 (t, *J* = 6.3 Hz, 3H) ppm; ^13^C NMR (75.4 MHz, CDCl_3_) δ 217.0, 193.6, 138.2, 136.6, 130.4, 128.1, 106.9, 79.6, 31.6, 28.9, 27.7, 22.5, 14.0 ppm; MS (*m*/*z*) 264 (M^+^ (^37^Cl), 0.76), 262 (M^+^ (^35^Cl), 2.08), 139 (100); IR (neat) 2927, 2857, 1931, 1654, 1590, 1460, 1397, 1274, 1091 cm^−1^; HRMS-EI (*m*/*z*) calcd for C_16_H_19_O^35^Cl^+^ [M^+^]: 262.1124; found: 262.1130.

#### 2-Hexyl-1-(4'-nitrophenyl)buta-2,3-dien-1-one (**2e**)

The reaction of 1,10-phenanthroline (5.5 mg, 0.03 mmol), 2,2'-bipyridine (4.7 mg, 0.03 mmol), CuCl (5.9 mg, 0.06 mmol), K_2_CO_3_ (20.8 mg, 0.15 mmol), dry toluene (1.5 mL), DBAD (13.9 mg, 0.06 mmol), and 2-hexyl-1-(4-nitrophenyl)buta-2,3-dien-1-ol (82.7 mg, 0.3 mmol)/dry toluene (1.5 mL) afforded **2e** (51.4 mg, 63%) (eluent: petroleum ether/diethyl ether = 20:1): liquid; ^1^H NMR (300 MHz, CDCl_3_) δ 8.24 (d, *J* = 8.7 Hz, 2H), 7.84 (d, *J* = 8.7 Hz, 2H), 5.12 (t, *J* = 2.9 Hz, 2H), 2.44–2.33 (m, 2H), 1.57–1.44 (m, 2H), 1.44–1.20 (m, 6H), 0.89 (t, *J* = 6.5 Hz, 3H) ppm; ^13^C NMR (75.4 MHz, CDCl_3_) δ 218.0, 193.4, 149.4, 143.6, 129.7, 123.1, 107.6, 80.4, 31.5, 28.8, 27.7, 27.3, 22.5, 14.0 ppm; MS (*m*/*z*) 273 (M^+^, 2.56), 150 (100); IR (neat) 2927, 2857, 1930, 1660, 1602, 1526, 1461, 1349, 1272, 1104, 1011 cm^−1^; HRMS-EI (*m*/*z*) calcd for C_16_H_19_NO_3_^+^ [M^+^]: 273.1365; found: 273.1367.

#### 2-Hexyl-1-(3-furanyl)buta-2,3-dien-1-one (**2f**)

The reaction of 1,10-phenanthroline (5.4 mg, 0.03 mmol), 2,2'-bipyridine (4.8 mg, 0.03 mmol), CuCl (6.2 mg, 0.06 mmol), K_2_CO_3_ (21.3 mg, 0.15 mmol), dry toluene (1.5 mL), DBAD (13.6 mg, 0.06 mmol), and 2-hexyl-1-(3-furanyl)buta-2,3-dien-1-ol (66.2 mg, 0.3 mmol)/dry toluene (1.5 mL) afforded **2f** (40.4 mg, 61%): liquid; ^1^H NMR (300 MHz, CDCl_3_) δ 8.10 (s, 1H), 7.37 (s, 1H), 6.81 (d, *J* = 1.2 Hz, 1H), 5.21 (t, *J* = 2.9 Hz, 2H), 2.39–2.27 (m, 2 H), 1.52–1.38 (m, 2H), 1.38–1.18 (m, 6H), 0.87 (t, *J* = 6.5 Hz, 3H) ppm; ^13^C NMR (75.4 MHz, CDCl_3_) δ 215.8, 186.4, 147.4, 143.0, 126.7, 110.0, 108.1, 80.0, 31.6, 28.9, 27.8, 27.6, 22.6, 14.1 ppm; MS (*m*/*z*) 218 (M^+^, 3.49), 95 (100); IR (neat) 2956, 2928, 2857, 1933, 1724, 1645, 1561, 1509, 1458, 1379, 1311, 1163, 1077, 1009 cm^−1^; HRMS-EI (*m*/*z*) calcd for C_14_H_18_O_2_^+^ [M^+^]: 218.1307; found: 218.1305.

#### 2-Pentyl-1-(3-thienyl)buta-2,3-dien-1-one (**2g**)

The reaction of 1,10-phenanthroline (5.5 mg, 0.03 mmol), 2,2'-bipyridine (4.8 mg, 0.03 mmol), CuCl (5.9 mg, 0.06 mmol), K_2_CO_3_ (20.9 mg, 0.15 mmol), dry toluene (1.5 mL), DBAD (13.7 mg, 0.06 mmol), and 2-pentyl-1-(3-thienyl)buta-2,3-dien-1-ol (66.4 mg, 0.3 mmol)/dry toluene (1.5 mL) afforded **2g** (48.3 mg, 73%) (eluent: petroleum ether/diethyl ether = 50:1): liquid; ^1^H NMR (300 MHz, CDCl_3_) δ 8.07 (d, *J* = 1.8 Hz, 1H) 7.53 (d, *J* = 4.8 Hz, 1H), 7.25 (dd, *J*_1_ = 4.8 Hz, *J*_2_ = 3.3 Hz, 1H), 5.16 (d, *J* = 2.7 Hz, 2H), 2.41–2.32 (m, 2H), 1.56–1.42 (m, 2H), 1.42–1.24 (m, 4H), 0.90 (t, *J* = 6.6 Hz, 3H) ppm; ^13^C NMR (75.4 MHz, CDCl_3_) δ 216.0, 187.1, 141.5, 132.2, 128.2, 125.1, 107.6, 79.6, 31.4, 27.9, 27.5, 22.4, 14.0 ppm; MS (*m*/*z*) 220 (M^+^, 3.02), 111 (100); IR (neat) 2956, 2927, 2861, 1933, 1641, 1511, 1460, 1411, 1260, 1082 cm^−1^; HRMS-EI (*m*/*z*) calcd for C_13_H_16_OS^+^ [M^+^]: 220.0922; found: 220.0922.

#### 2-Methyl-1-(1-naphthyl)buta-2,3-dien-1-one (**2h**)

The reaction of 1,10-phenanthroline (5.5 mg, 0.03 mmol), 2,2'-bipyridine (4.8 mg, 0.03 mmol), CuCl (6.2 mg, 0.06 mmol), K_2_CO_3_ (21.4 mg, 0.15 mmol), dry toluene (1.5 mL), DBAD (13.5 mg, 0.06 mmol), and 2-methyl-1-naphthylbuta-2,3-dien-1-ol (63.6 mg, 0.3 mmol)/dry toluene (1.5 mL) afforded **2h** (47.2 mg, 74%, an unknown substance could not be separated via column chromatography and the purity of **2h** is 95%, which was determined by ^1^H NMR with mesitylene as the internal standard): liquid; ^1^H NMR (300 MHz, CDCl_3_) δ 8.11–8.03 (m, 1H), 7.95–7.81 (m, 2H), 7.61–7.39 (m, 4H), 4.80 (q, *J* = 2.8 Hz, 2H), 2.11 (t, *J* = 2.7 Hz, 3H) ppm; ^13^C NMR (75.4 MHz, CDCl_3_) δ 218.6, 197.5, 136.8, 133.5, 130.6, 130.4, 128.2, 126.9, 126.5, 126.1, 125.3, 123.9, 104.8, 78.2, 13.8 ppm; MS (*m*/*z*) 208 (M^+^, 63.16), 155 (100); IR (neat) 3059, 1957, 1930, 1650, 1508, 1285, 1251, 1204, 1155, 1080, 1059 cm^−1^; HRMS-EI (*m*/*z*) calcd for C_15_H_12_O [M^+^]: 208.0888; found: 208.0887.

#### 2-Allyl-1-phenylbuta-2,3-dien-1-one (**2i**)

The reaction of 1,10-phenanthroline (5.4 mg, 0.03 mmol), 2,2'-bipyridine (4.6 mg, 0.03 mmol), CuCl (6.1 mg, 0.06 mmol), K_2_CO_3_ (21.5 mg, 0.15 mmol), dry toluene (1.5 mL), DBAD (13.7 mg, 0.06 mmol), and 2-allyl-1-phenylbuta-2,3-dien-1-ol (55.1 mg, 0.3 mmol)/dry toluene (1.5 mL) afforded **2i** (41.1 mg, 75%) (eluent: petroleum ether/diethyl ether = 40:1): liquid; ^1^H NMR (300 MHz, CDCl_3_) δ 7.78 (d, *J* = 7.8 Hz, 2H), 7.50 (t, *J* = 7.2 Hz, 1H), 7.39 (t, *J* = 7.5 Hz, 2H), 5.98–5.82 (m, 1H), 5.22–5.04 (m, 4H), 3.20–3.14 (m, 2H) ppm; ^13^C NMR (75.4 MHz, CDCl_3_) δ 217.2, 194.1, 138.0, 134.9, 132.1, 129.0, 127.8, 116.4, 105.3, 79.8, 32.5 ppm; MS (*m*/*z*) 184 (M^+^, 2.53), 105 (100); IR (neat) 3081, 3062, 2982, 1956, 1931, 1651, 1598, 1578, 1447, 1422, 1316, 1272 cm^−1^; HRMS-EI (*m*/*z*) calcd for C_13_H_12_O [M^+^]: 184.0888; found: 184.0889.

#### 2-Butyl-1-phenylnona-2,3-dien-1-one (**2j**)

The reaction of 1,10-phenanthroline (5.6 mg, 0.03 mmol), 2,2'-bipyridine (4.9 mg, 0.03 mmol), CuCl (6.2 mg, 0.06 mmol), K_2_CO_3_ (21.5 mg, 0.15 mmol), dry toluene (1.5 mL), DBAD (14.1 mg, 0.06 mmol), and 2-butyl-1-phenylnona-2,3-dien-1-ol (82.2 mg, 0.3 mmol)/dry toluene (1.5 mL) afforded **2j** [43] (74.8 mg, 91%): liquid; ^1^H NMR (300 MHz, CDCl_3_) 7.71 (d, *J* = 7.8 Hz, 2H), 7.50–7.42 (m, 1H), 7.40–7.32 (m, 2H), 5.36 (t, *J* = 7.2 Hz, 1H), 2.48–2.30 (m, 2H), 2.16–1.96 (m, 2H), 1.55–1.11 (m, 10H), 0.93 (t, *J* = 6.9 Hz, 3H), 0.84 (t, *J* = 7.2 Hz, 3H) ppm; ^13^C NMR (75.4 MHz, CDCl_3_) δ 213.3, 195.7, 138.9, 131.5, 128.8, 127.6, 107.4, 95.0, 31.1, 30.2, 28.6, 28.4, 27.8, 22.31, 22.29, 13.89, 13.86 ppm.

#### 2-Propyl-1-phenylbuta-2,3-dien-1-one (**2k**)

The reaction of 1,10-phenanthroline (49.1 mg, 0.27 mmol), 2,2'-bipyridine (42.6 mg, 0.27 mmol), CuCl (54.2 mg, 0.54 mmol), K_2_CO_3_ (373.8 mg, 2.7 mmol), dry toluene (9 mL), DBAD (124.4 mg, 0.54 mmol), and 2-propyl-1-phenylbuta-2,3-dien-1-ol (1.0141 g, 5.4 mmol)/dry toluene (9 mL) afforded **2k** (0.7512 g, 74%): liquid; ^1^H NMR (300 MHz, CDCl_3_) δ 7.76 (d, *J* = 7.5 Hz, 2H), 7.50 (t, *J* = 7.4 Hz, 1H), 7.39 (t, *J* = 7.5 Hz, 2H), 5.05 (s, 2H), 2.39 (t, *J* = 7.2 Hz, 2H), 1.62–1.46 (m, 2H), 0.99 (t, *J* = 7.5 Hz, 3H) ppm; ^13^C NMR (75.4 MHz, CDCl_3_) δ 217.1, 194.9, 138.3, 131.9, 129.0, 127.8, 106.7, 79.3, 29.9, 21.1, 13.7 ppm; MS (*m*/*z*) 186 (M^+^, 6.46), 105 (100); IR (neat) 2961, 2932, 2872, 1933, 1651, 1598, 1578, 1447, 1315, 1271 cm^−1^; HRMS-EI (*m*/*z*) calcd for C_13_H_14_O^+^ [M^+^]: 186.1045; found: 186.1045.

### General experimental procedure for the oxidation of allenic alcohols with pure oxygen

#### 3-Hexylocta-1,2-dien-4-one (**2l**)

Typical procedure: 1,10-phenanthroline (6.9 mg, 0.0375 mmol), 2,2'-bipyridine (5.8 mg, 0.0375 mmol), CuCl (5.9 mg, 0.06 mmol), and K_2_CO_3_ (20.9 mg, 0.15 mmol) were added sequentially to an oven dried Schlenk tube, which was purged with air and refilled with oxygen (twice). Then 1.5 mL of dry toluene was added, the resulting mixture was stirred at rt for 0.5 h which was followed by the sequential addition of DBAD (14.0 mg, 0.06 mmol), 2-hexyl-1-butylbuta-2,3-dien-1-ol (63.8 mg, 0.3 mmol) and 1.5 mL of dry toluene. After stirring at 60 °C for 24 h, the reaction mixture was filtered through silica gel (100–140 mesh) and washed with diethyl ether. Evaporation of the solvent and flash chromatography on silica gel (eluent: petroleum ether/ether = 50:1) afforded **2l** (37.1 mg, 58%) (conv. = 81%, yield = 72% (based on the alcohol consumed)): liquid; ^1^H NMR (300 MHz, CDCl_3_) δ 5.14 (d, *J* = 2.7 Hz, 2H), 2.62 (t, *J* = 7.4 Hz, 2H), 2.18–2.09 (m, 2H), 1.60–1.47 (m, 2H), 1.42–1.18 (m, 10H), 0.92–0.78 (m, 6H) ppm; ^13^C NMR (75.4 MHz, CDCl_3_) δ 216.2, 201.3, 108.5, 79.3, 38.9, 31.6, 28.8, 27.8, 27.2, 26.2, 22.5, 22.3, 14.0, 13.8 ppm; MS (*m*/*z*) 208 (M^+^, 1.00), 85 (100); IR (neat) 2958, 2929, 2862, 1934, 1679, 1461, 1174 cm^−1^; HRMS-EI (*m*/*z*) calcd for C_14_H_24_O^+^ [M^+^]: 208.1827; found: 208.1828.

#### 4-Pentyl-1-phenylhexa-4,5-dien-3-one (**2m**)

The reaction of 1,10-phenanthroline (6.9 mg, 0.0375 mmol), 2,2'-bipyridine (5.9 mg, 0.0375 mmol), CuCl (6.1 mg, 0.06 mmol), K_2_CO_3_ (21.4 mg, 0.15 mmol), dry toluene (1.5 mL), DBAD (13.9 mg, 0.06 mmol), and 2-pentyl-1-(phenylethyl)buta-2,3-dien-1-ol (72.7 mg, 0.3 mmol)/dry toluene (1.5 mL) afforded **2m** (43.8 mg, 60%) (conv. = 81%, yield = 74% (based on the alcohol consumed)): liquid; ^1^H NMR (300 MHz, CDCl_3_) δ 7.31–7.22 (m, 2H), 7.22–7.13 (m, 3H), 5.13 (s, 2 H), 3.02–2.85 (m, 4H), 2.22–2.10 (m, 2H), 1.45–1.20 (m, 6H), 0.88 (t, *J* = 6.5 Hz, 3H) ppm; ^13^C NMR (75.4 MHz, CDCl_3_) δ 216.3, 200.1, 141.3, 128.3, 126.0, 108.6, 79.7, 40.9, 31.4, 30.9, 27.4, 26.1, 22.4, 14.0 ppm; MS (*m*/*z*) 242 (M^+^, 0.87), 105 (100); IR (neat) 2956, 2928, 2861, 1933, 1678, 1496, 1456, 1171, 1100 cm^−1^; Anal. calcd for C_17_H_22_O: C, 84.25; H, 9.15. found: C, 84.16; H, 9.50.

## Supporting Information

File 1 ^1^H and ^13^C NMR spectra of products prepared.
