# Supplementary material for: CuCl-catalyzed aerobic oxidation of 2,3-allenols to 1,2-allenic ketones with 1:1 combination of phenanthroline and bipyridine as ligands
Source: Beilstein J Org Chem. 2011 Apr 7;7:396–403. doi: 10.3762/bjoc.7.51 (PMC3079115; doi:10.3762/bjoc.7.51)
Supplement: File 1 — 1H and 13C NMR spectra of products prepared. [file Beilstein_J_Org_Chem-07-396-s001.pdf]

Supporting Information

for

CuCl-catalyzed aerobic oxidation of 2,3-allenols to 1,2-allenic ketones with 1:1 combination of phenanthroline and bipyridine as ligands

Shuxu Gao[1], Yu Liu[1], and Shengming Ma*[1,2]

Address: [1]Shanghai Key Laboratory of Green Chemistry and Chemical Processes, Department of Chemistry, East China Normal University, 3663 North Zhongshan Road, Shanghai 200062, P. R. China and [2]State Key Laboratory of Organometallic Chemistry, Shanghai Institute of Organic Chemistry, Chinese Academy of Sciences, 345 Lingling Lu, Shanghai 200032, P. R. China. Fax: (+86)-21-6260-9305

Email: Shengming Ma* - masm@sioc.ac.cn

* Corresponding author

^{1}H and ^{13}C NMR spectra of products prepared

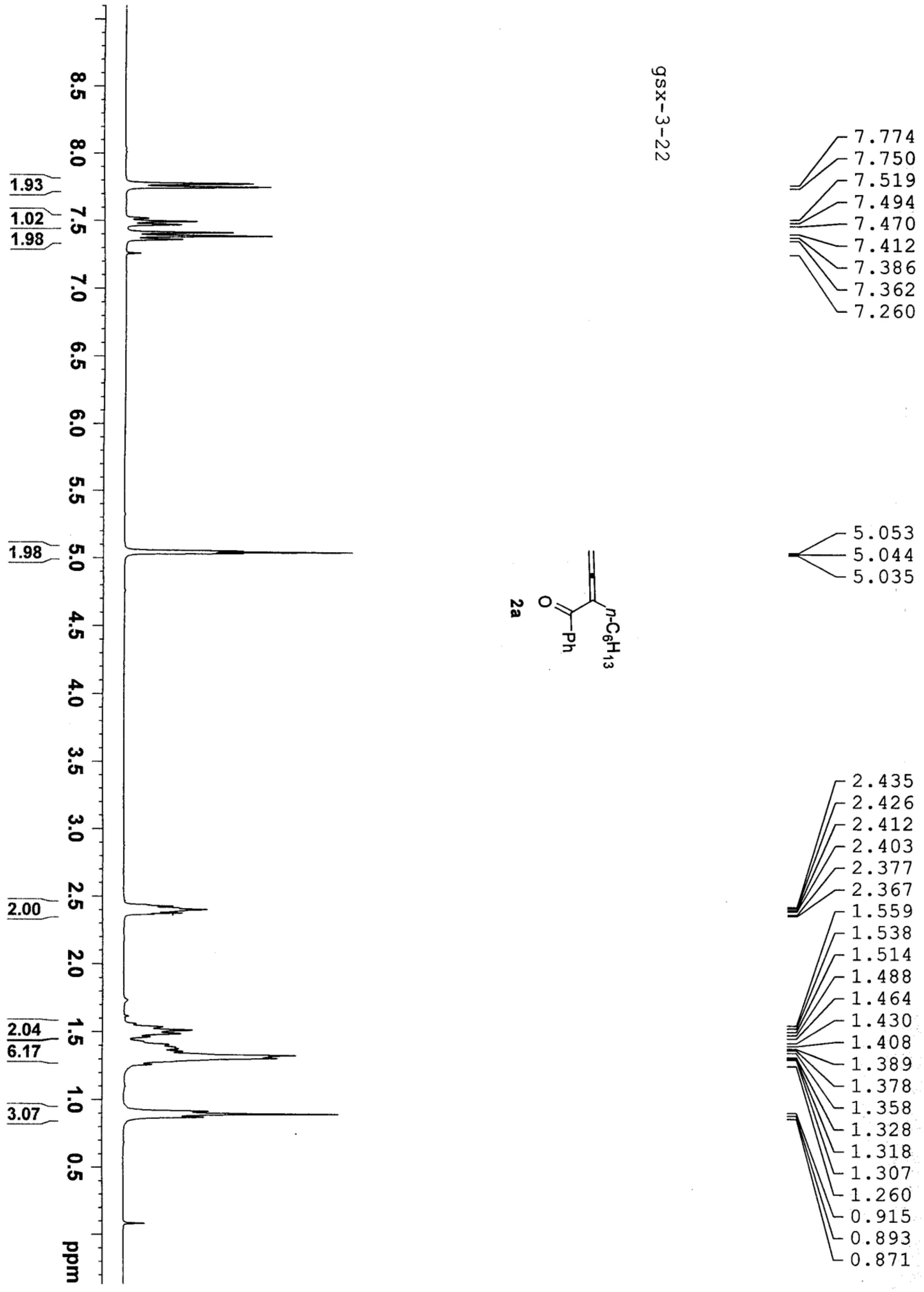
gsx-3-22
7.774
7.750
7.519
7.494
7.470
7.412
7.386
7.362
7.260
5.053
5.044
5.035
2.435
2.426
2.412
2.403
2.377
2.367
1.559
1.538
1.514
1.488
1.464
1.430
1.408
1.389
1.378
1.358
1.328
1.318
1.307
1.260
0.915
0.893
0.871
O
Ph
n-C6H13
2a
1.93
1.02
1.98
1.98
2.00
2.04
6.17
3.07
8.5
8.0
7.5
7.0
6.5
6.0
5.5
5.0
4.5
4.0
3.5
3.0
2.5
2.0
1.5
1.0
0.5
ppm

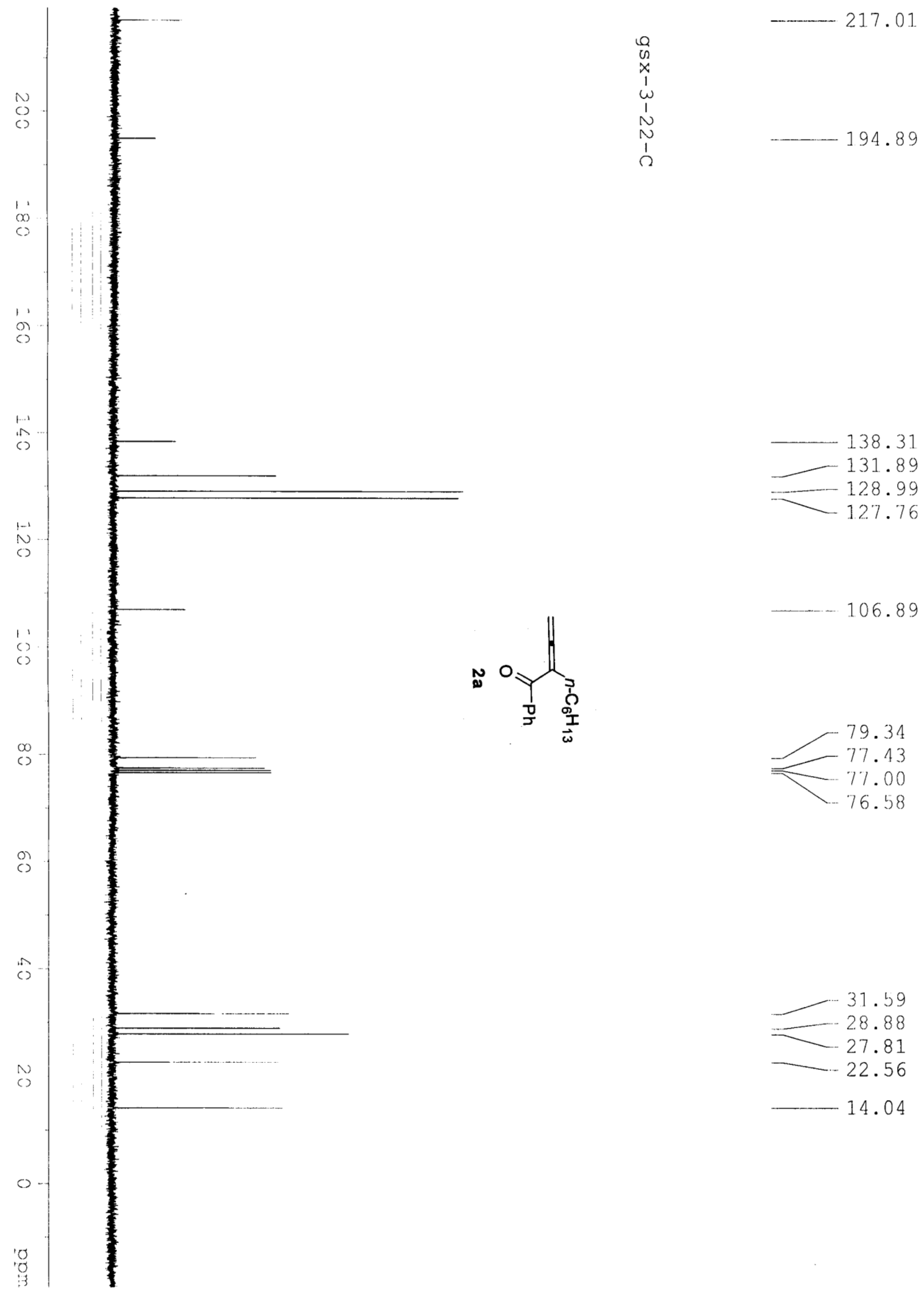
gsx-3-22-C
217.01
194.89
138.31
131.89
128.99
127.76
106.89
79.34
77.43
77.00
76.58
31.59
28.88
27.81
22.56
14.04
O
Ph
n-C6H13
2a
200
180
160
140
120
100
80
60
40
20
0
ppm

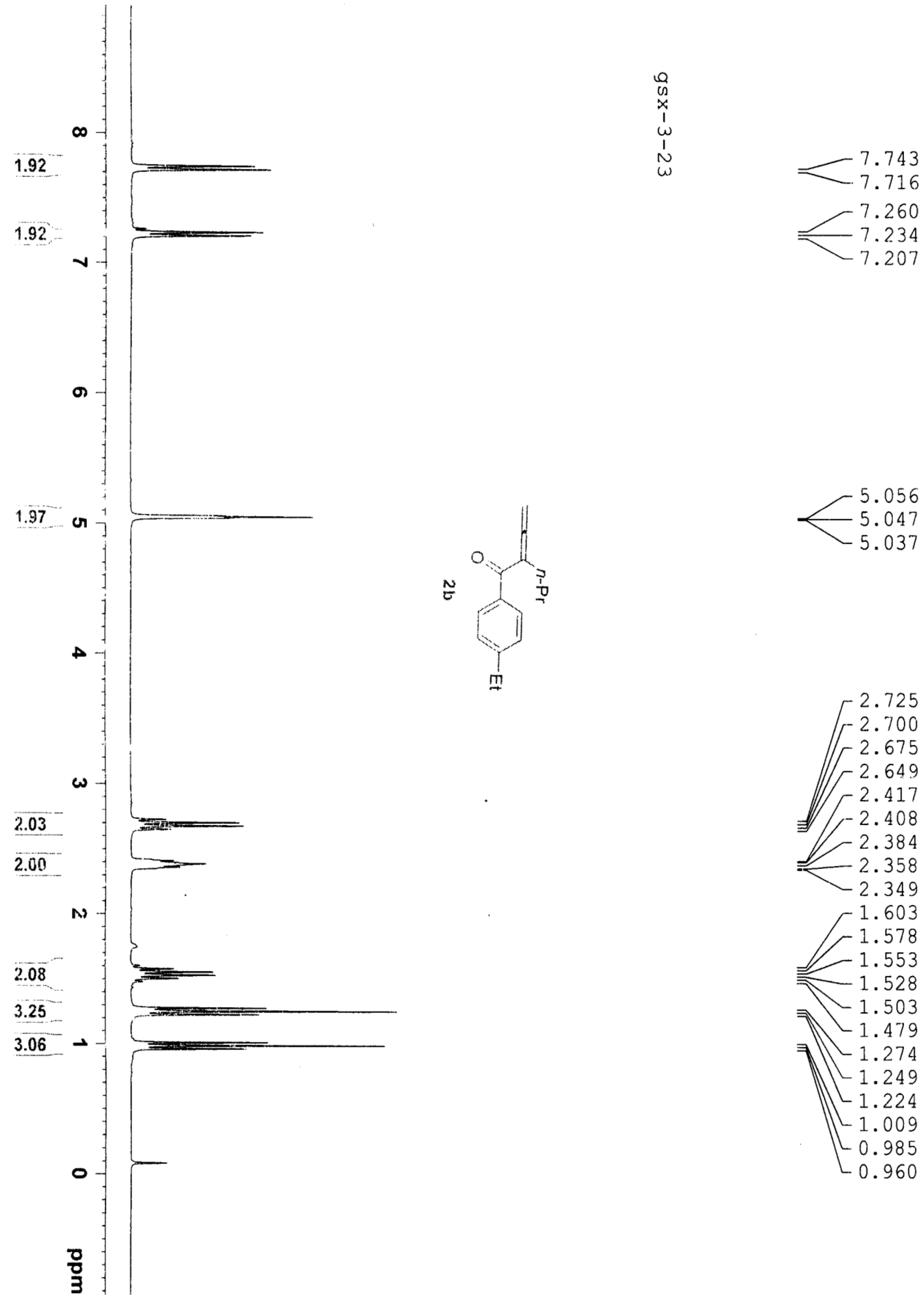
gsx-3-23
7.743
7.716
7.260
7.234
7.207
5.056
5.047
5.037
2.725
2.700
2.675
2.649
2.417
2.408
2.384
2.358
2.349
1.603
1.578
1.553
1.528
1.503
1.479
1.274
1.249
1.224
1.009
0.985
0.960
O
n-Pr
Et
2b
1.92
1.92
1.97
2.03
2.00
2.08
3.25
3.06
8
7
6
5
4
3
2
1
0
ppm

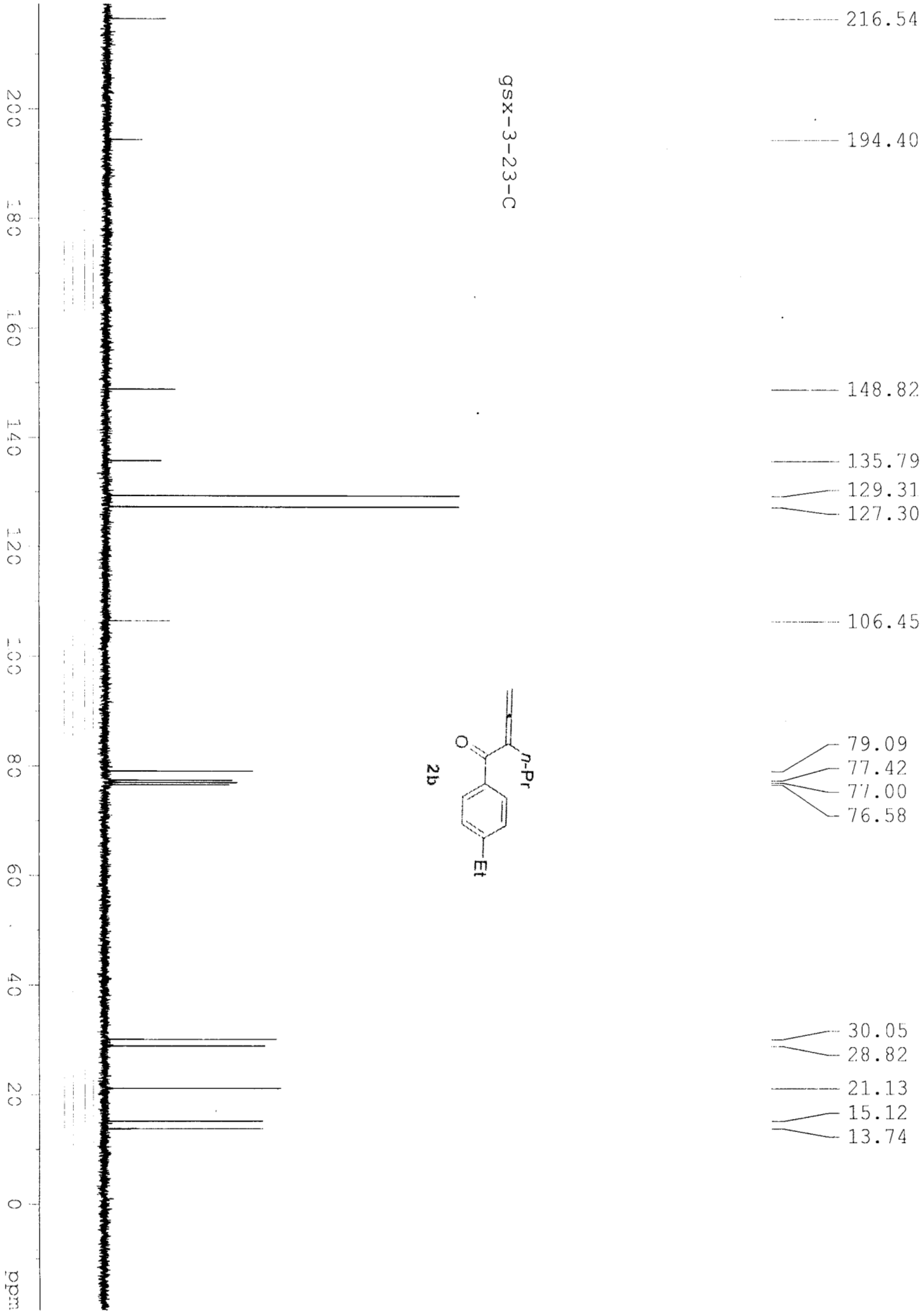
gsx-3-23-C
216.54
194.40
148.82
135.79
129.31
127.30
106.45
79.09
77.42
77.00
76.58
30.05
28.82
21.13
15.12
13.74
O
n-Pr
Et
2b
200
180
160
140
120
100
80
60
40
20
0
ppm

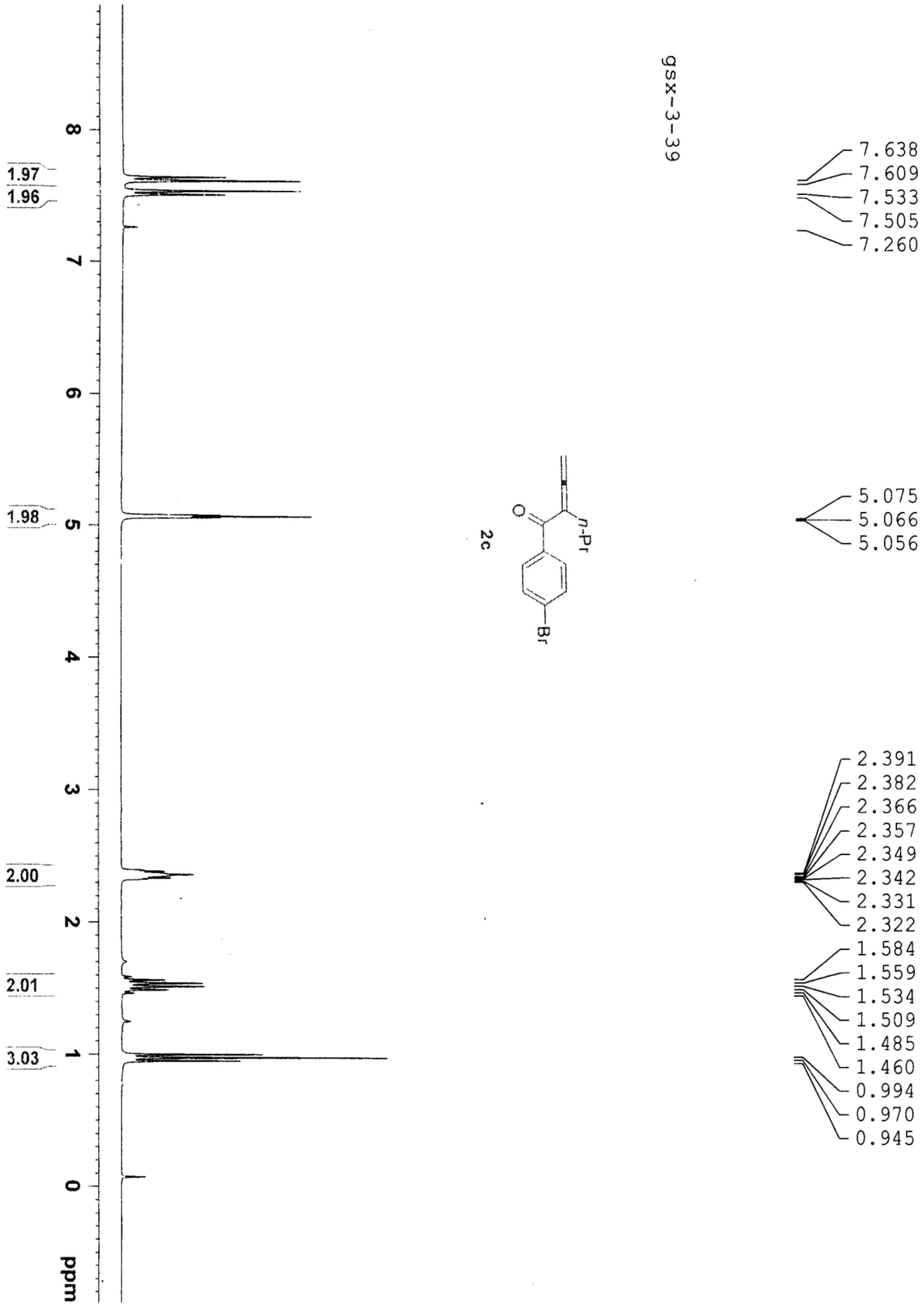
gsx-3-39
7.638
7.609
7.533
7.505
7.260
5.075
5.066
5.056
2.391
2.382
2.366
2.357
2.349
2.342
2.331
2.322
1.584
1.559
1.534
1.509
1.485
1.460
0.994
0.970
0.945
n-Pr
Br
2c
1.97
1.96
1.98
2.00
2.01
3.03
8
7
6
5
4
3
2
1
0
ppm

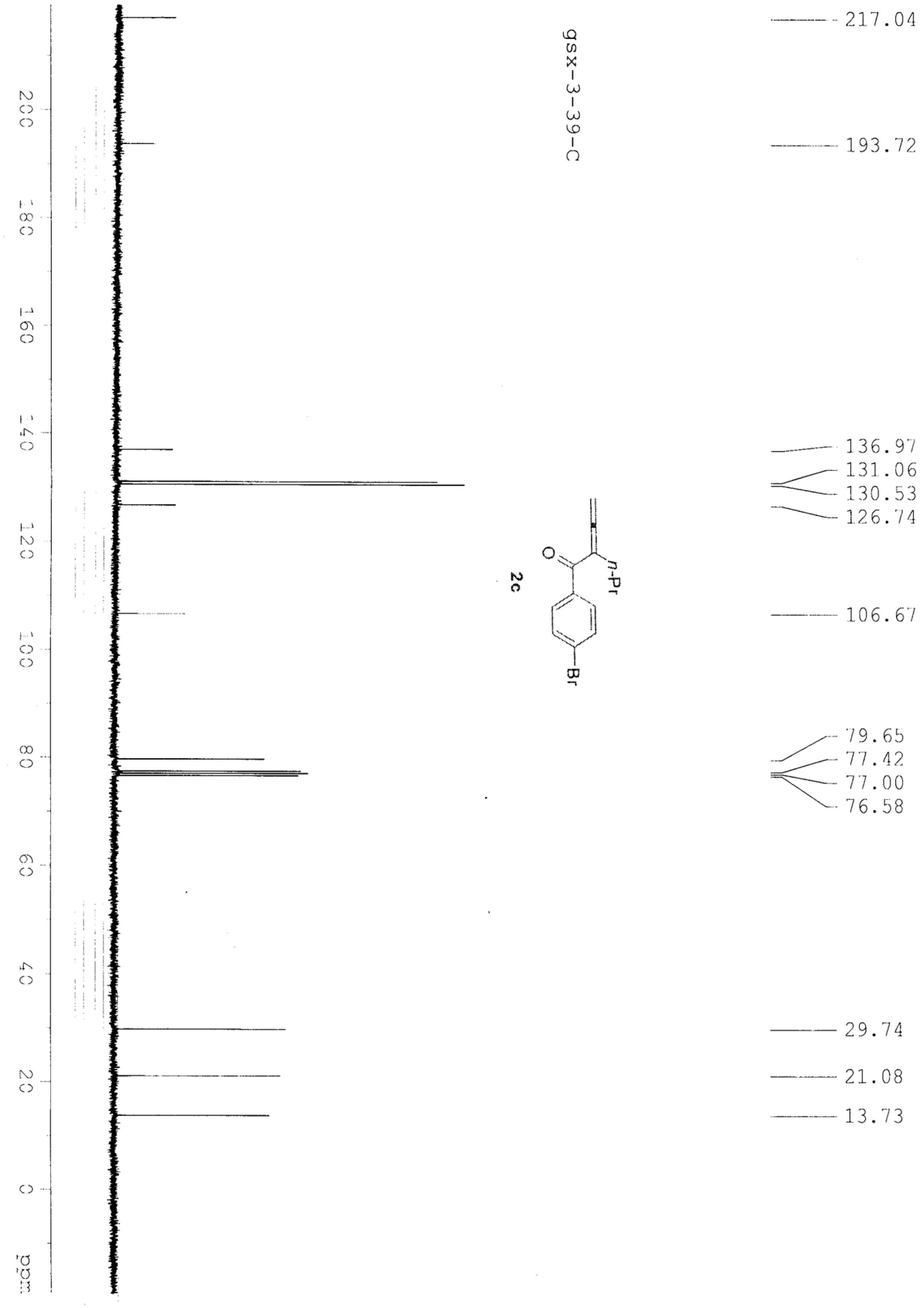
gsx-3-39-C
217.04
193.72
136.97
131.06
130.53
126.74
106.67
79.65
77.42
77.00
76.58
29.74
21.08
13.73
O
n-Pr
Br
2c
200
180
160
140
120
100
80
60
40
20
0
ppm

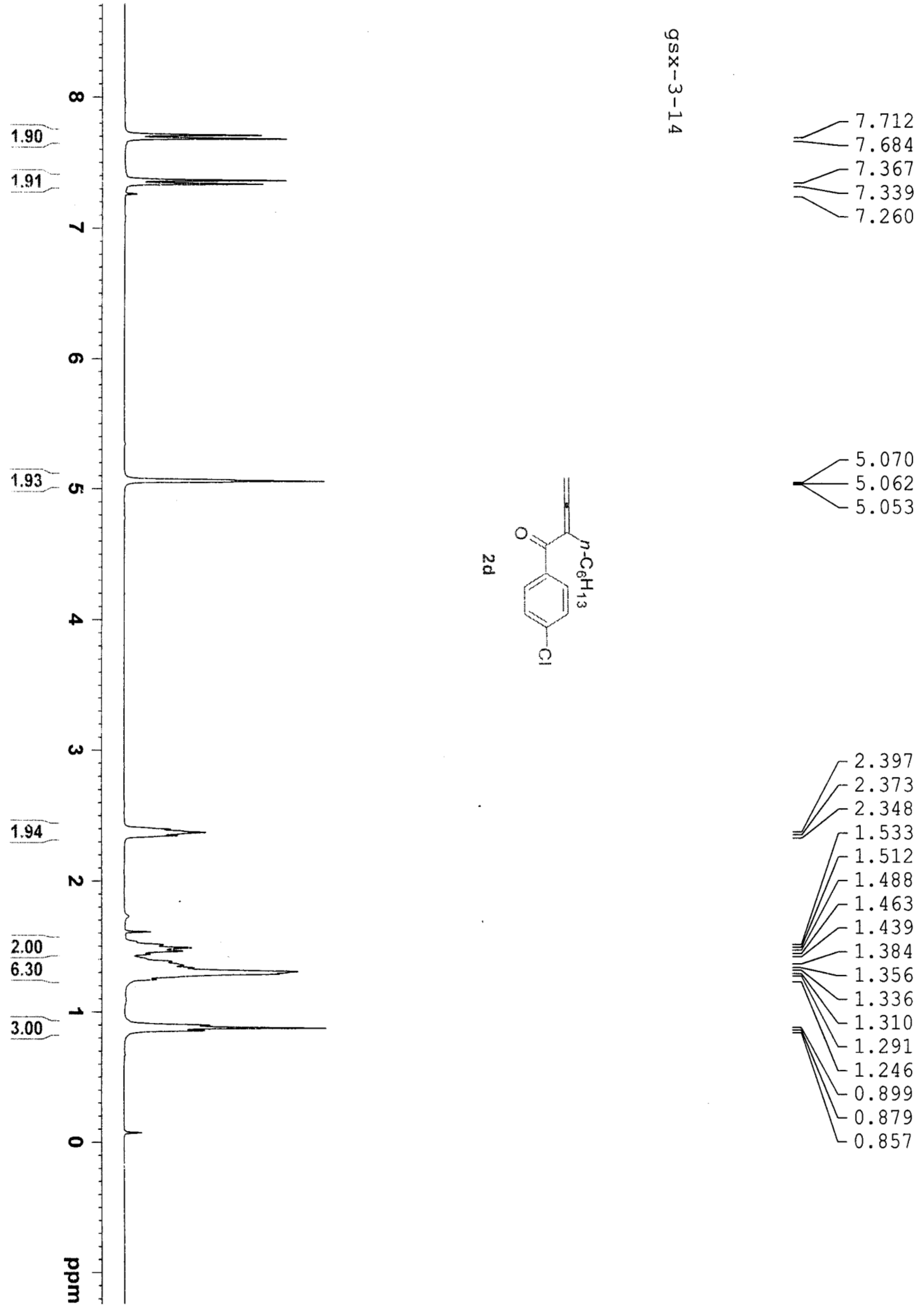
gsx-3-14
7.712
7.684
7.367
7.339
7.260
5.070
5.062
5.053
2.397
2.373
2.348
1.533
1.512
1.488
1.463
1.439
1.384
1.356
1.336
1.310
1.291
1.246
0.899
0.879
0.857
1.90
1.91
1.93
1.94
2.00
6.30
3.00
ppm
2d
n-C6H13
O
Cl

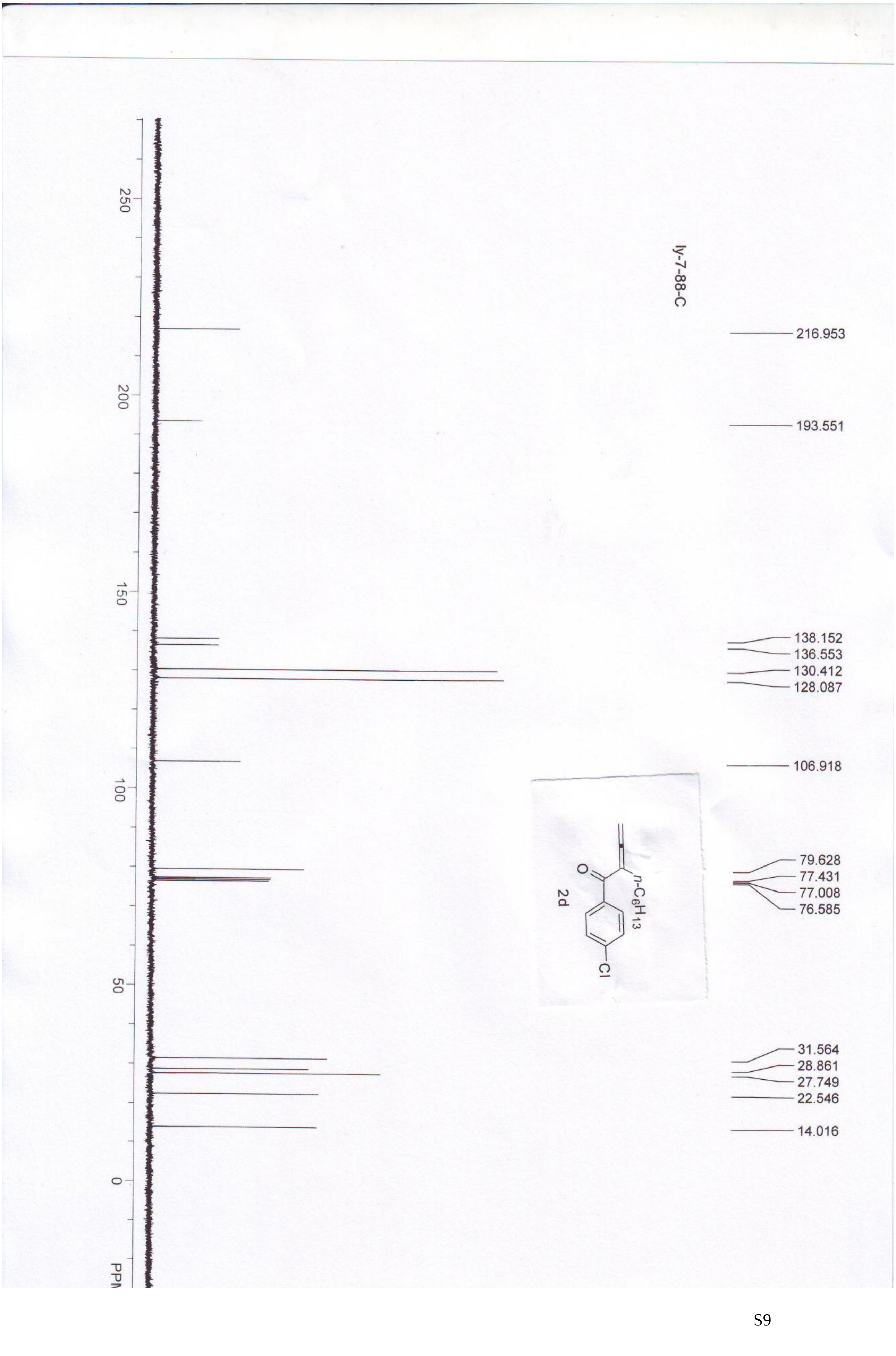
ly-7-88-C
216.953
193.551
138.152
136.553
130.412
128.087
106.918
79.628
77.431
77.008
76.585
31.564
28.861
27.749
22.546
14.016
O
n-C6H13
Cl
2d
250
200
150
100
50
0
PPM

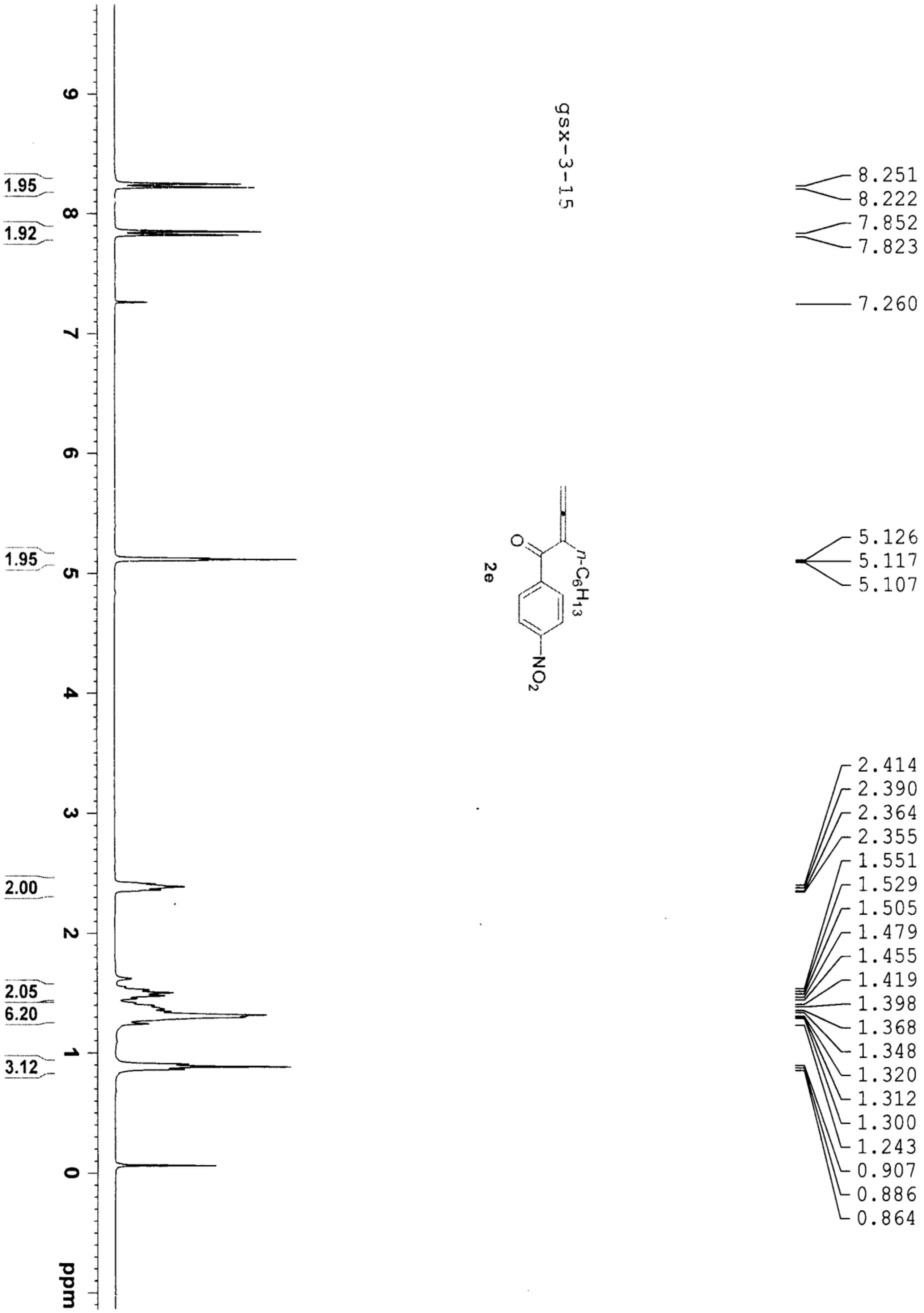
gsx-3-15
2e
n-C6H13
NO2
8.251
8.222
7.852
7.823
7.260
5.126
5.117
5.107
2.414
2.390
2.364
2.355
1.551
1.529
1.505
1.479
1.455
1.419
1.398
1.368
1.348
1.320
1.312
1.300
1.243
0.907
0.886
0.864
1.95
1.92
1.95
2.00
2.05
6.20
3.12
9
8
7
6
5
4
3
2
1
0
ppm

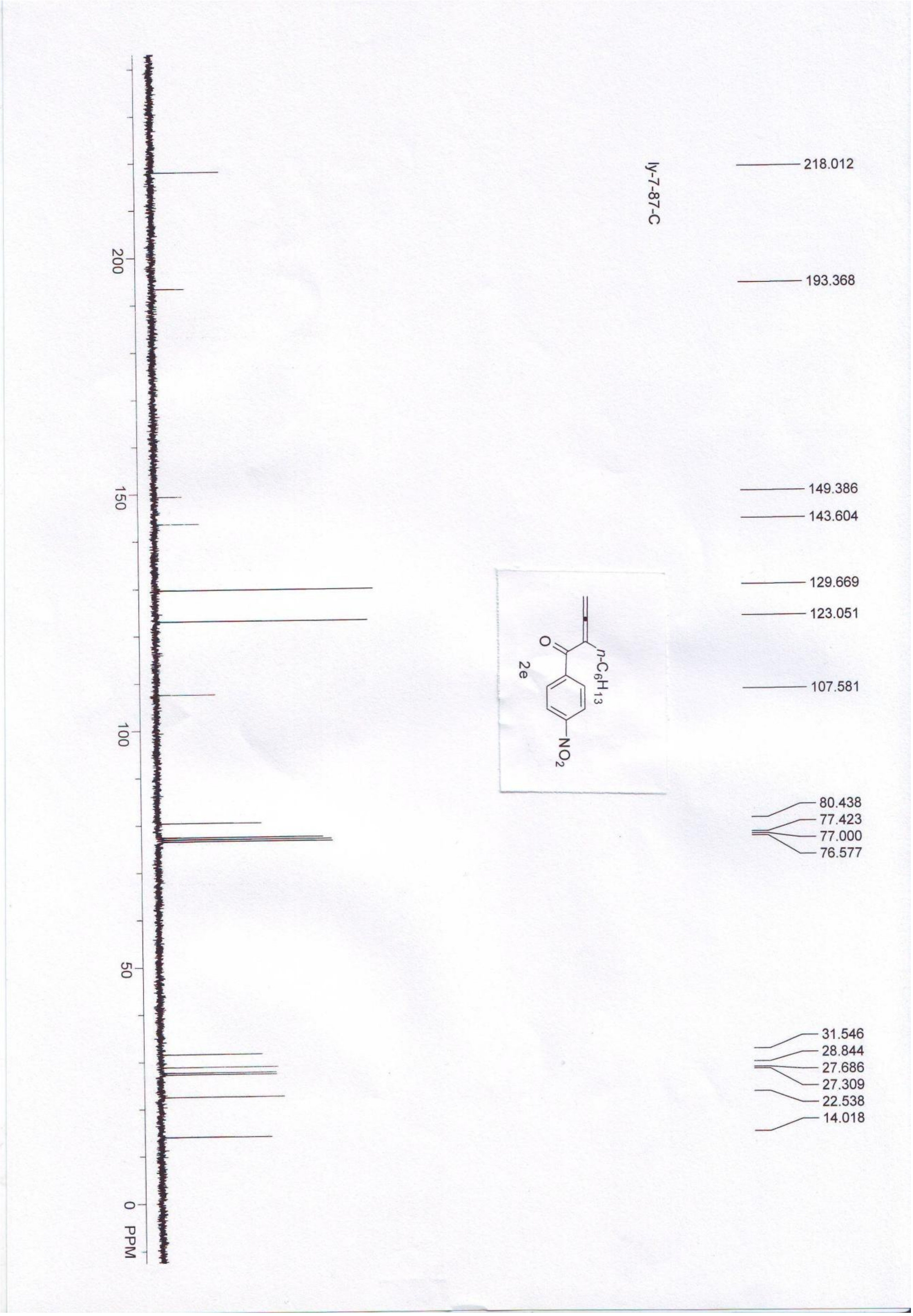
ly-7-87-C
218.012
193.368
149.386
143.604
129.669
123.051
107.581
80.438
77.423
77.000
76.577
31.546
28.844
27.686
27.309
22.538
14.018
O
n-C6H13
2e
NO2
200
150
100
50
0 PPM

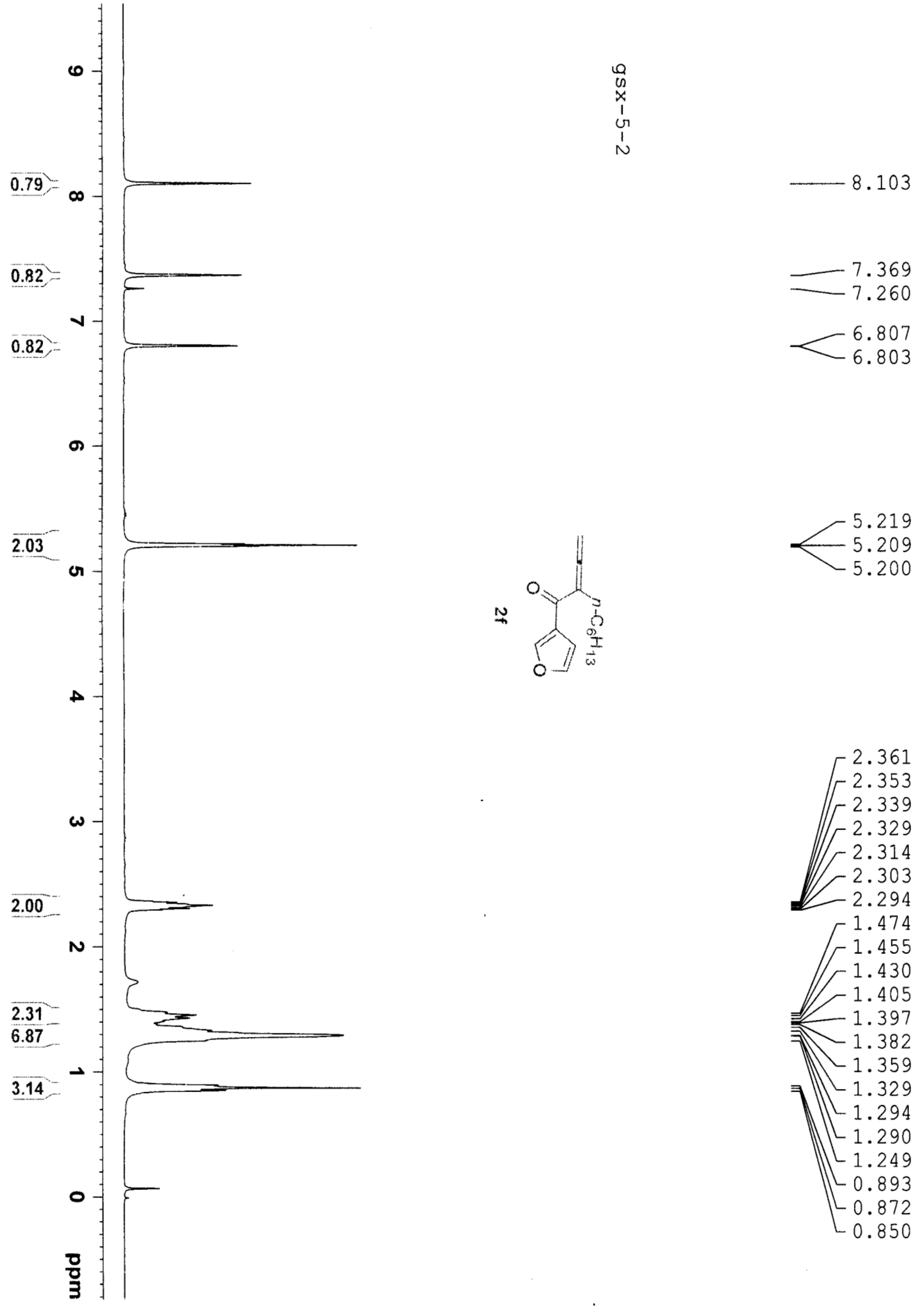
gsx-5-2
8.103
7.369
7.260
6.807
6.803
5.219
5.209
5.200
2.361
2.353
2.339
2.329
2.314
2.303
2.294
1.474
1.455
1.430
1.405
1.397
1.382
1.359
1.329
1.294
1.290
1.249
0.893
0.872
0.850
2f
n-C6H13
0.79
0.82
0.82
2.03
2.00
2.31
6.87
3.14
9
8
7
6
5
4
3
2
1
0
ppm

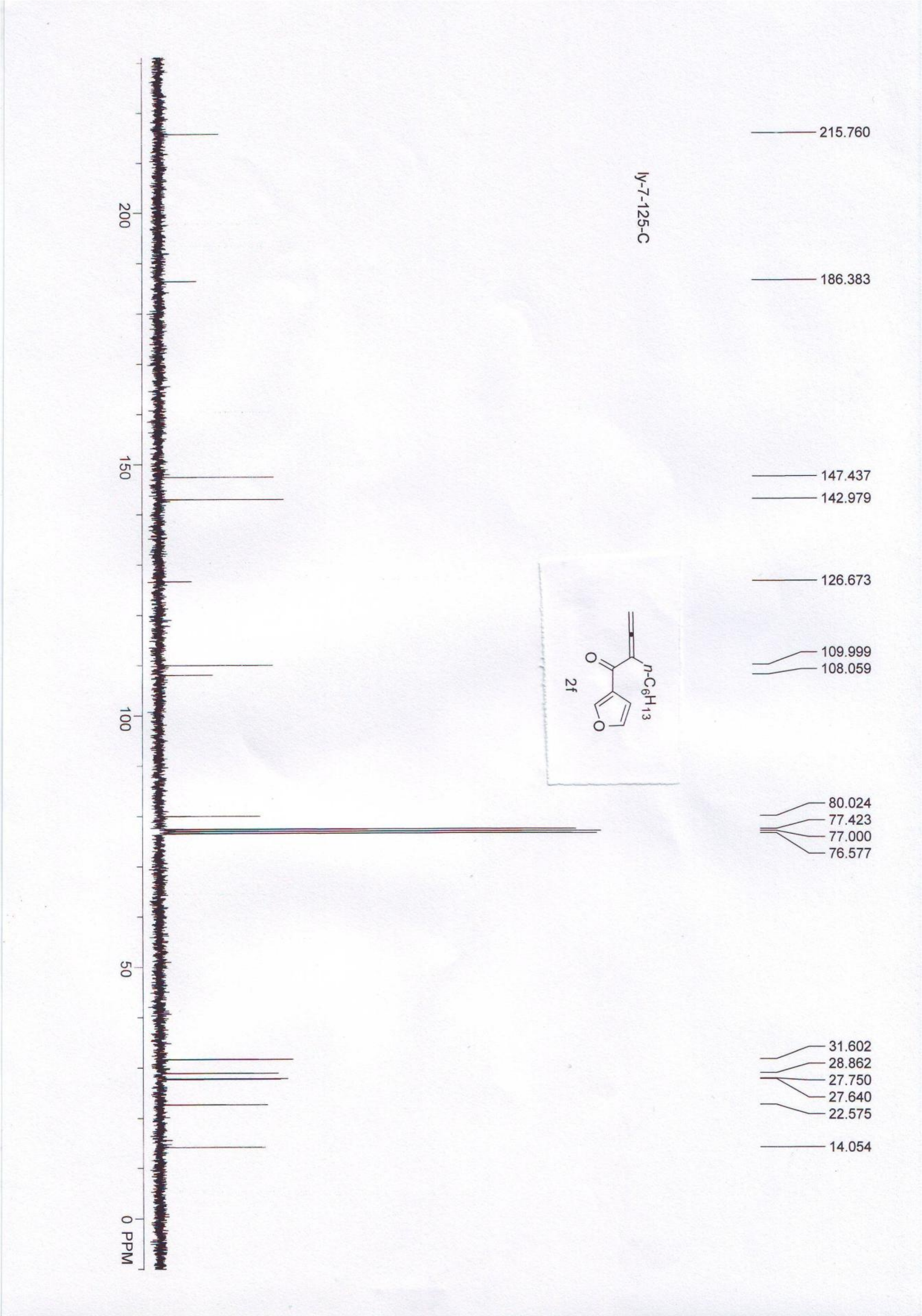
ly-7-125-C
215.760
186.383
147.437
142.979
126.673
109.999
108.059
80.024
77.423
77.000
76.577
31.602
28.862
27.750
27.640
22.575
14.054
n-C6H13
2f
200
150
100
50
0 PPM

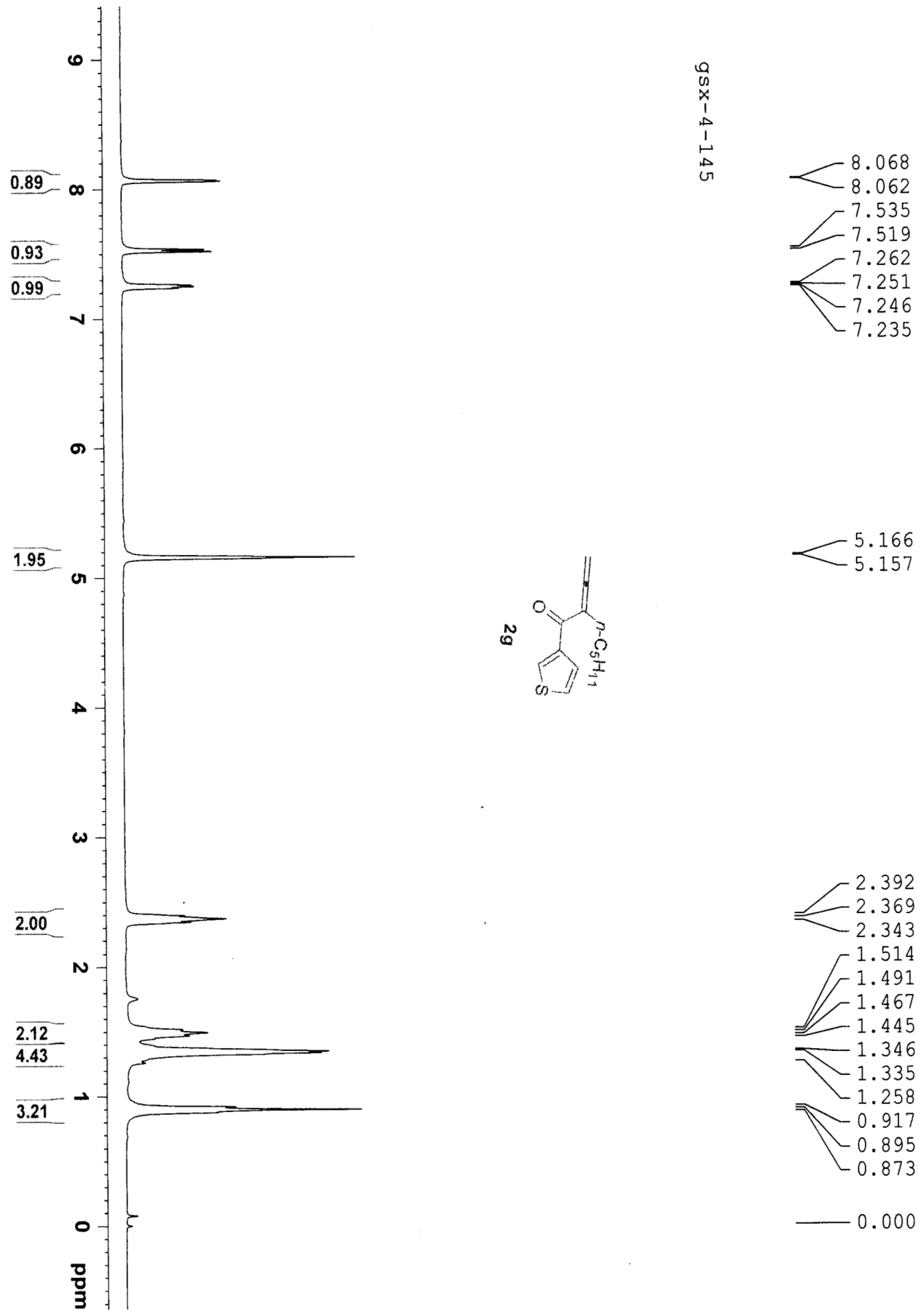
gsx-4-145
8.068
8.062
7.535
7.519
7.262
7.251
7.246
7.235
5.166
5.157
2.392
2.369
2.343
1.514
1.491
1.467
1.445
1.346
1.335
1.258
0.917
0.895
0.873
0.000
n-C5H11
O
S
2g
9
8
7
6
5
4
3
2
1
0
ppm
0.89
0.93
0.99
1.95
2.00
2.12
4.43
3.21

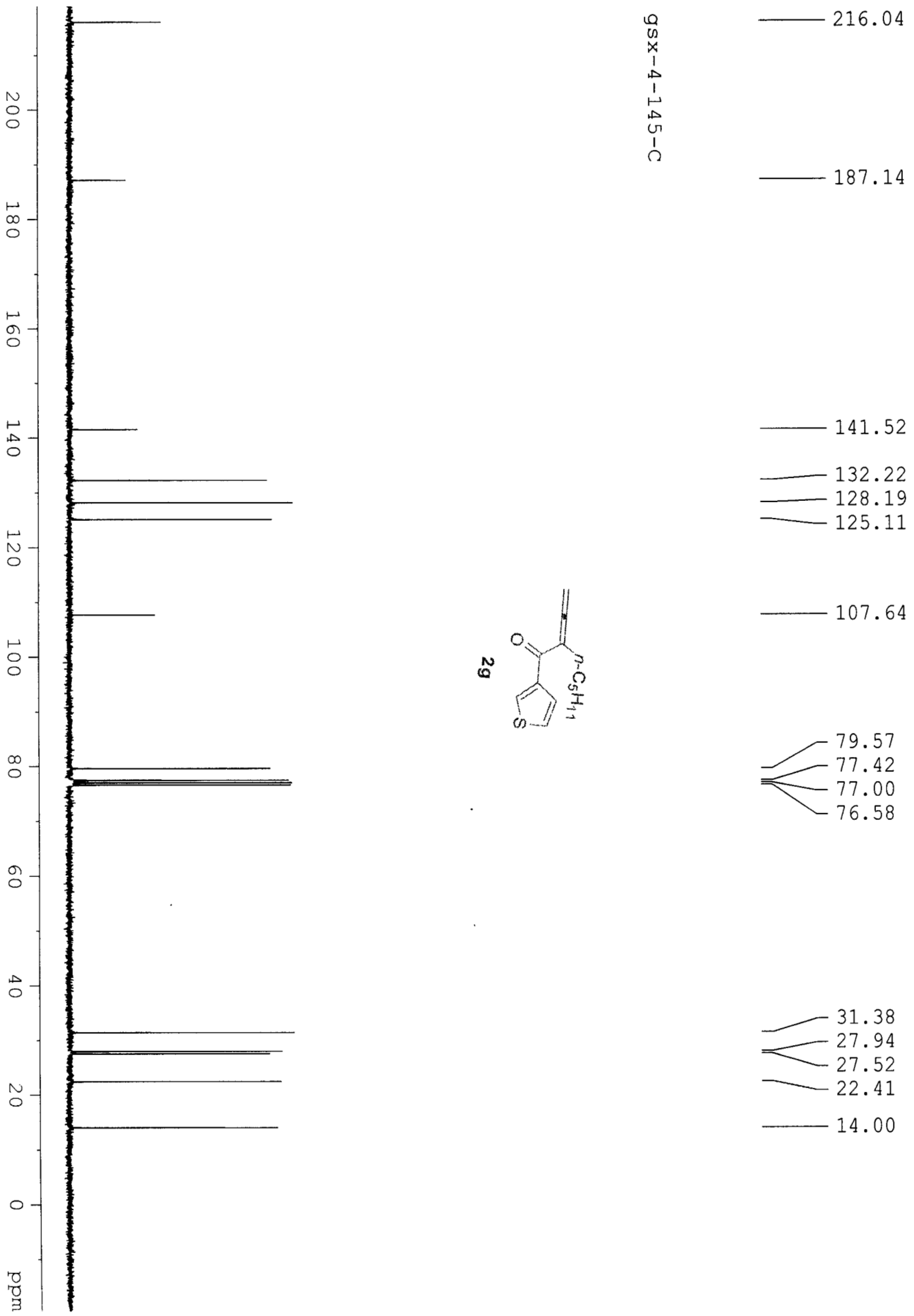
gsx-4-145-C
216.04
187.14
141.52
132.22
128.19
125.11
107.64
79.57
77.42
77.00
76.58
31.38
27.94
27.52
22.41
14.00
2g
n-C5H11
ppm
200
180
160
140
120
100
80
60
40
20
0

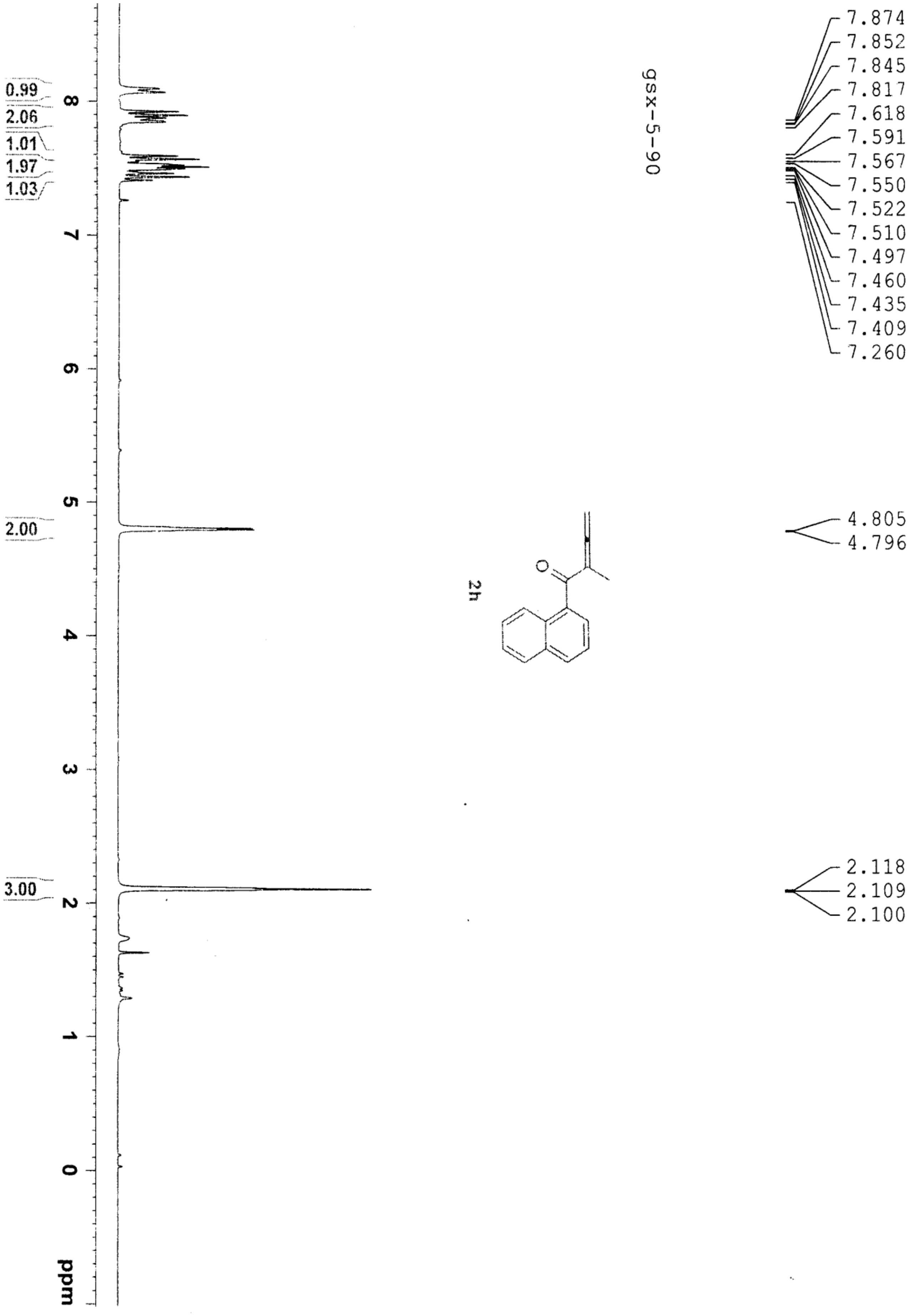

gsx-5-90
2h
7.874
7.852
7.845
7.817
7.618
7.591
7.567
7.550
7.522
7.510
7.497
7.460
7.435
7.409
7.260
4.805
4.796
2.118
2.109
2.100
0.99
2.06
1.01
1.97
1.03
2.00
3.00
ppm

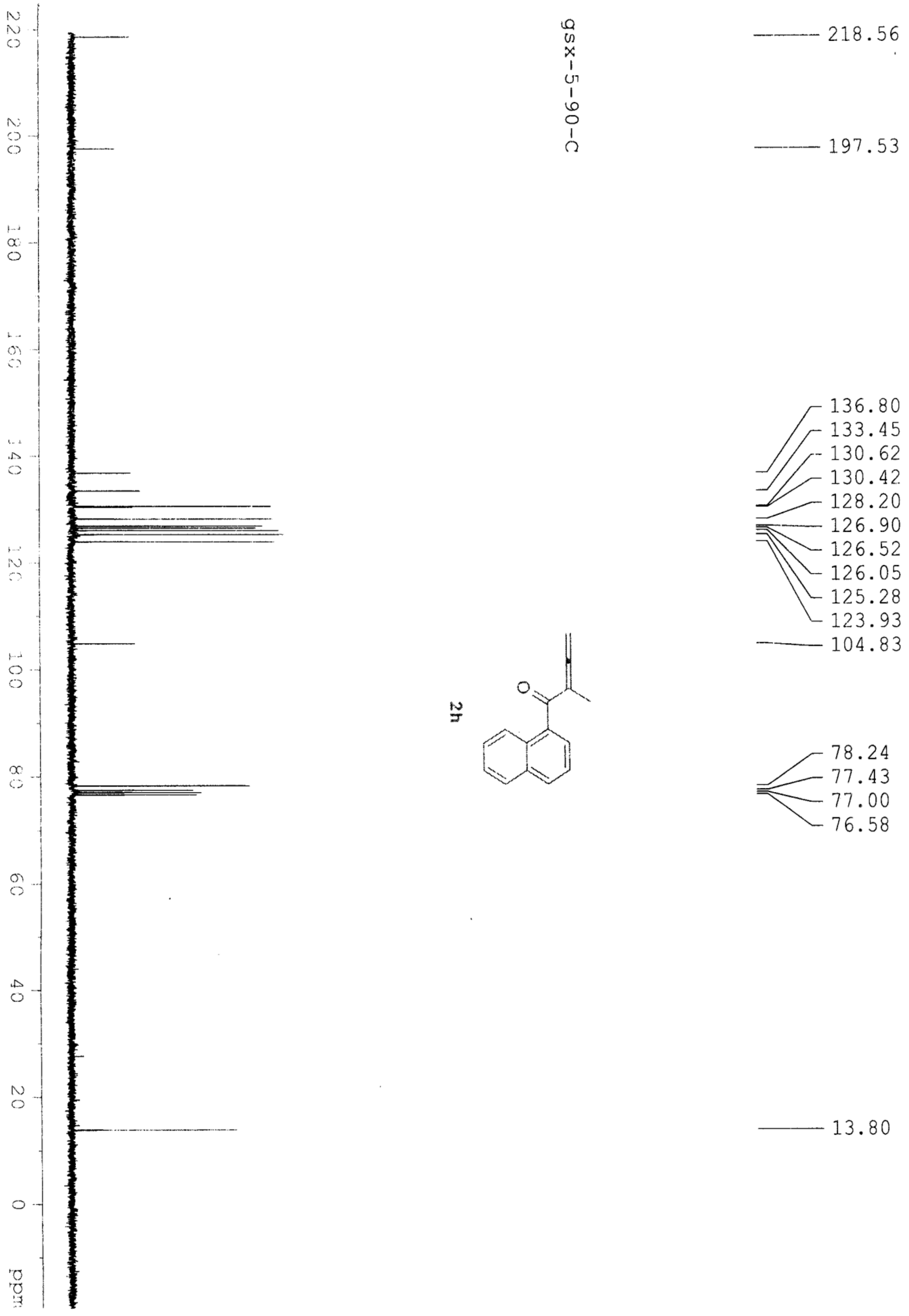
gsx-5-90-C
218.56
197.53
136.80
133.45
130.62
130.42
128.20
126.90
126.52
126.05
125.28
123.93
104.83
78.24
77.43
77.00
76.58
13.80
2h
220
200
180
160
140
120
100
80
60
40
20
0
ppm

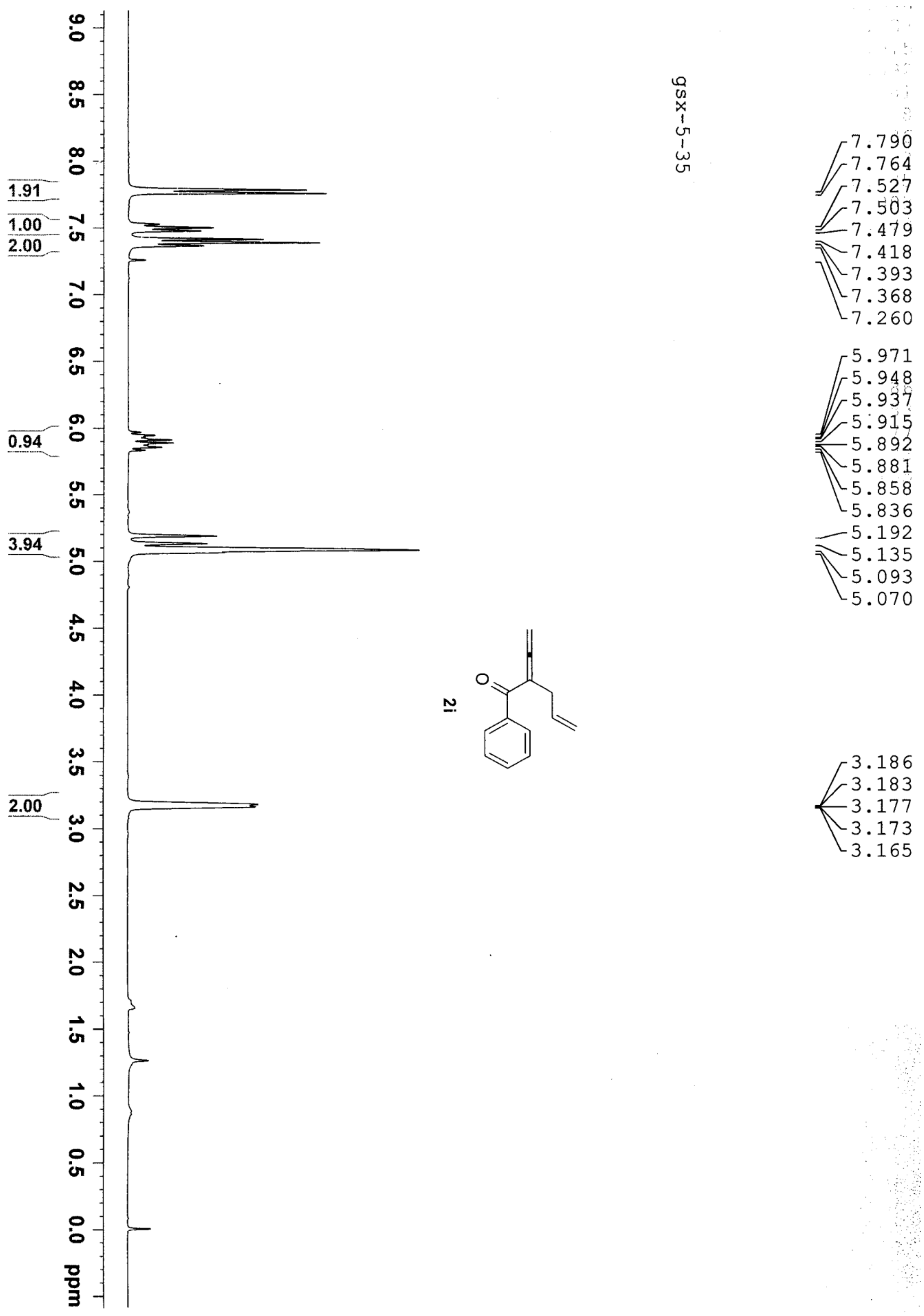
gsx-5-35
7.790
7.764
7.527
7.503
7.479
7.418
7.393
7.368
7.260
5.971
5.948
5.937
5.915
5.892
5.881
5.858
5.836
5.192
5.135
5.093
5.070
3.186
3.183
3.177
3.173
3.165
2i
1.91
1.00
2.00
0.94
3.94
2.00
9.0
8.5
8.0
7.5
7.0
6.5
6.0
5.5
5.0
4.5
4.0
3.5
3.0
2.5
2.0
1.5
1.0
0.5
0.0
ppm

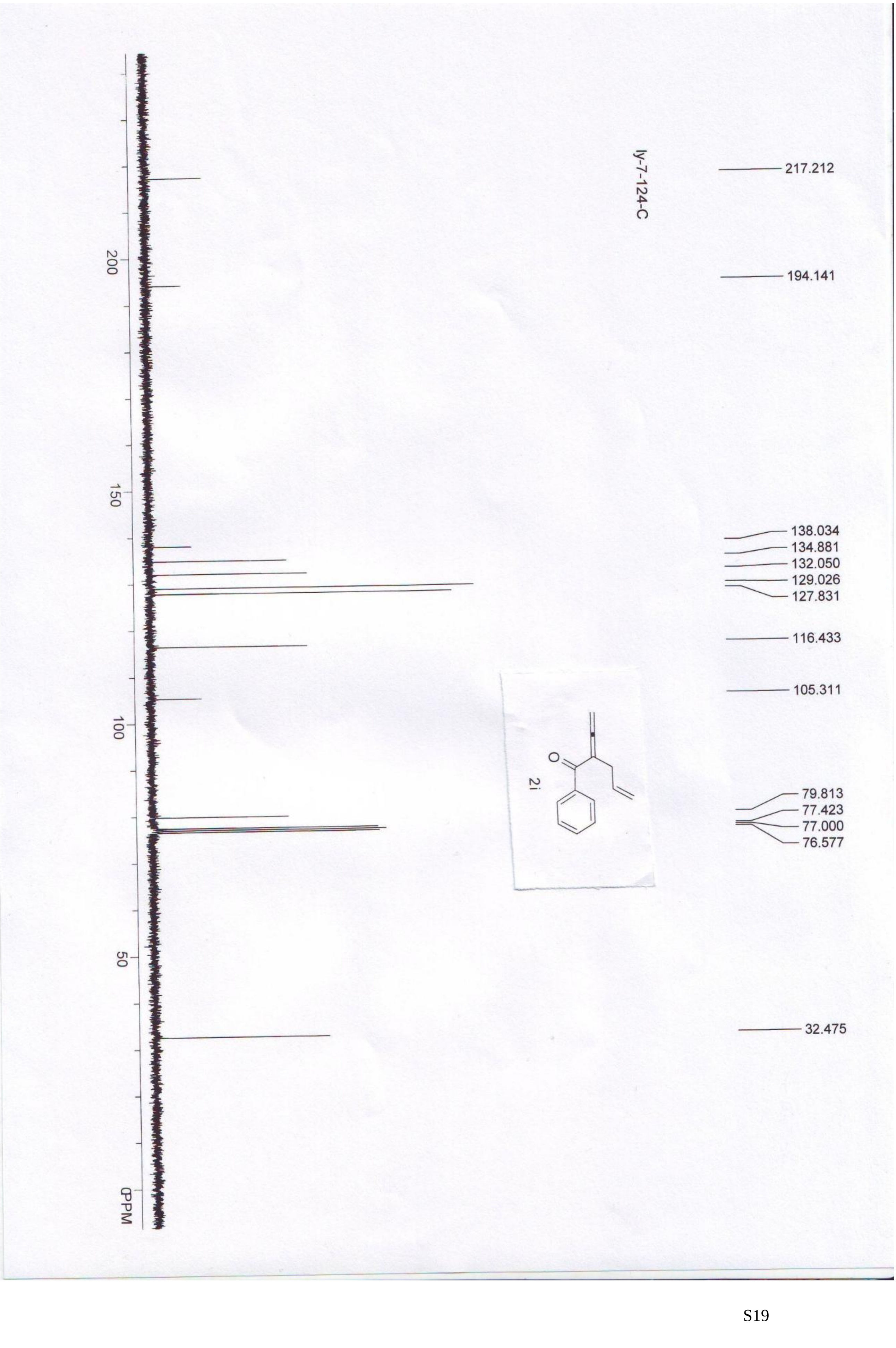
ly-7-124-C
217.212
194.141
138.034
134.881
132.050
129.026
127.831
116.433
105.311
79.813
77.423
77.000
76.577
32.475
2i
200
150
100
50
0PPM

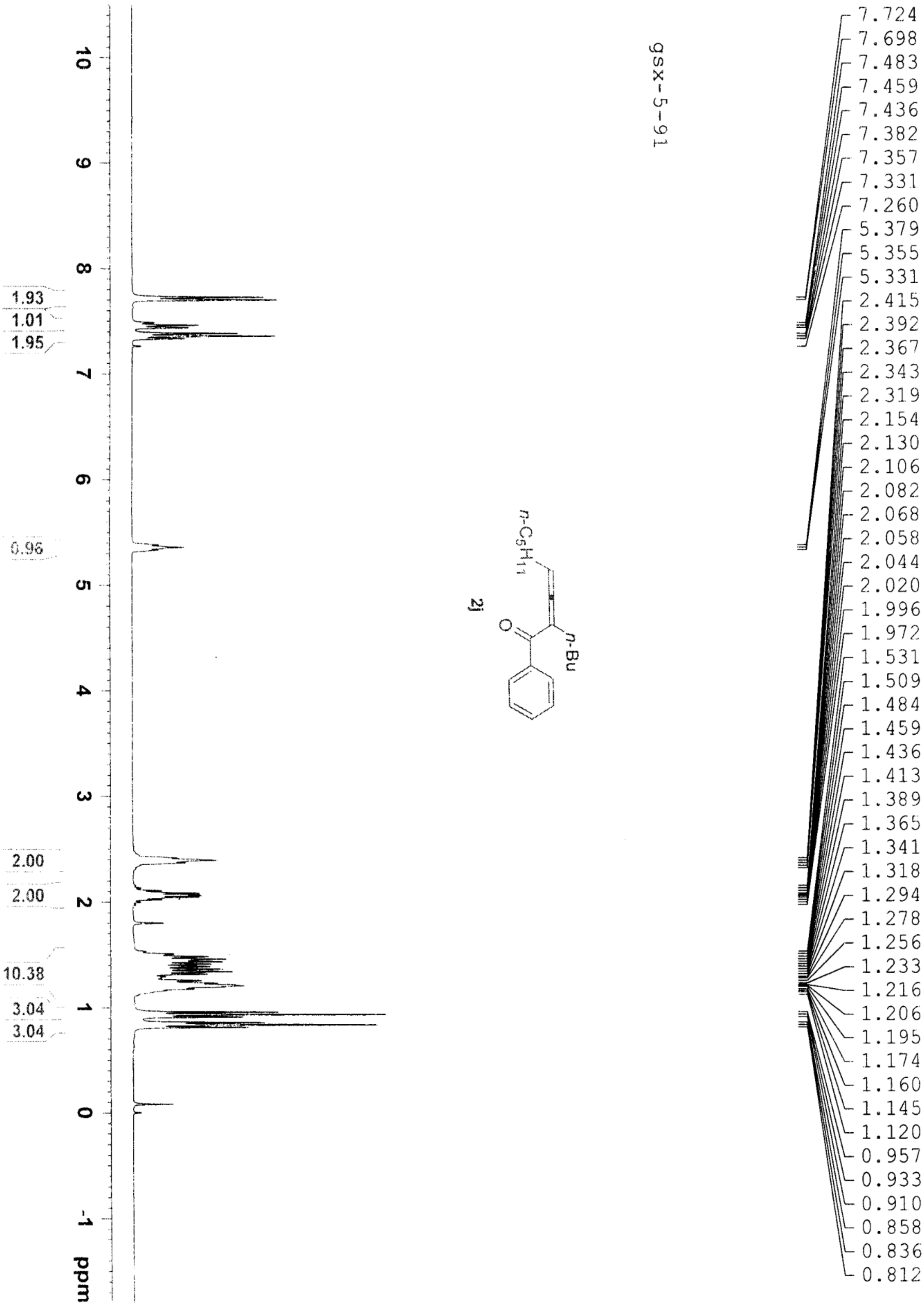
gsx-5-91
7.724
7.698
7.483
7.459
7.436
7.382
7.357
7.331
7.260
5.379
5.355
5.331
2.415
2.392
2.367
2.343
2.319
2.154
2.130
2.106
2.082
2.068
2.058
2.044
2.020
1.996
1.972
1.531
1.509
1.484
1.459
1.436
1.413
1.389
1.365
1.341
1.318
1.294
1.278
1.256
1.233
1.216
1.206
1.195
1.174
1.160
1.145
1.120
0.957
0.933
0.910
0.858
0.836
0.812
n-C5H11
n-Bu
O
2j
1.93
1.01
1.95
0.96
2.00
2.00
10.38
3.04
3.04
10
9
8
7
6
5
4
3
2
1
0
-1
ppm

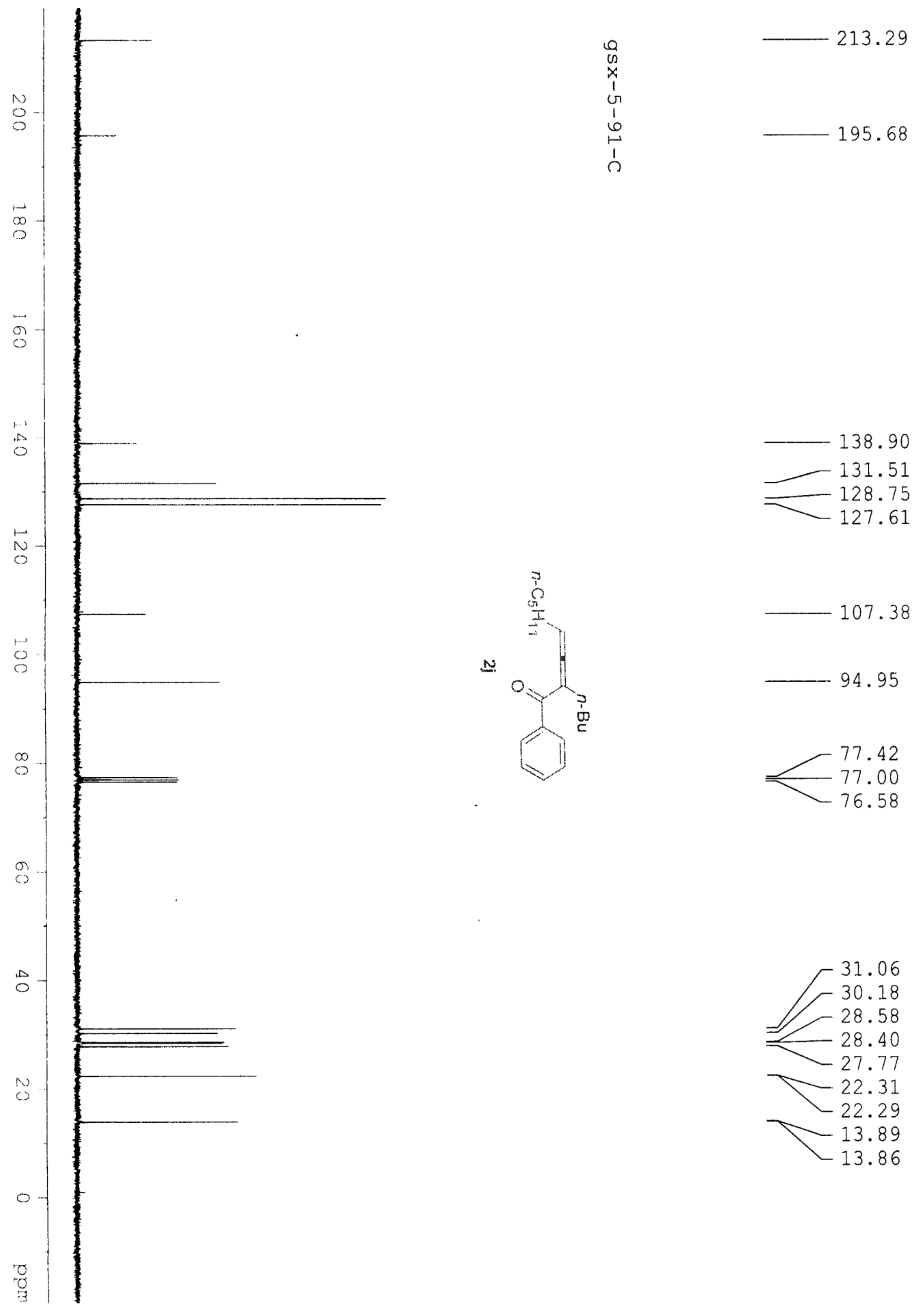
gsx-5-91-C
213.29
195.68
138.90
131.51
128.75
127.61
107.38
94.95
77.42
77.00
76.58
31.06
30.18
28.58
28.40
27.77
22.31
22.29
13.89
13.86
n-C5H11
n-Bu
O
2j
200
180
160
140
120
100
80
60
40
20
0
ppm

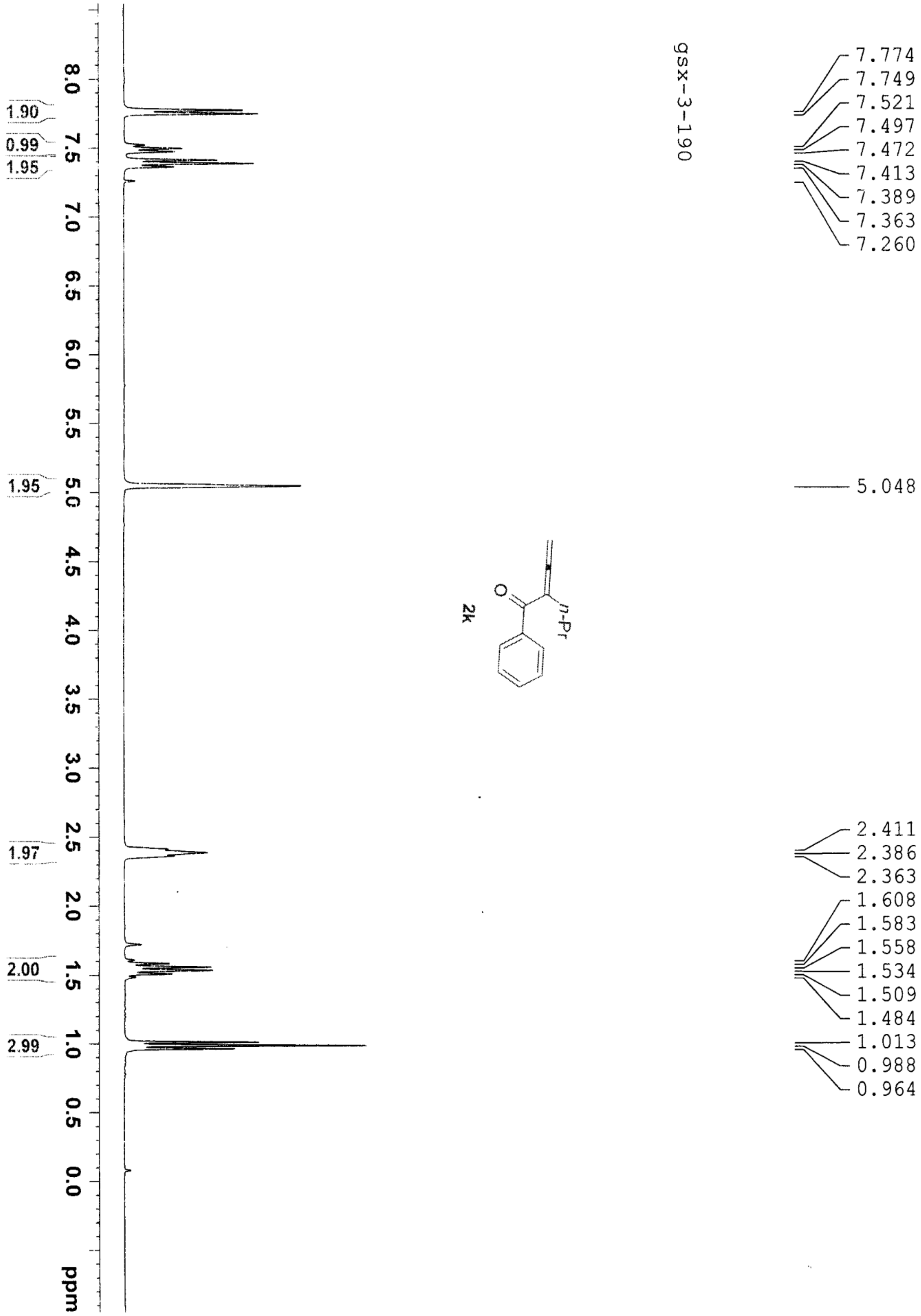
gsx-3-190
7.774
7.749
7.521
7.497
7.472
7.413
7.389
7.363
7.260
5.048
2.411
2.386
2.363
1.608
1.583
1.558
1.534
1.509
1.484
1.013
0.988
0.964
2k
n-Pr
O
1.90
0.99
1.95
1.95
1.97
2.00
2.99
8.0
7.5
7.0
6.5
6.0
5.5
5.0
4.5
4.0
3.5
3.0
2.5
2.0
1.5
1.0
0.5
0.0
ppm

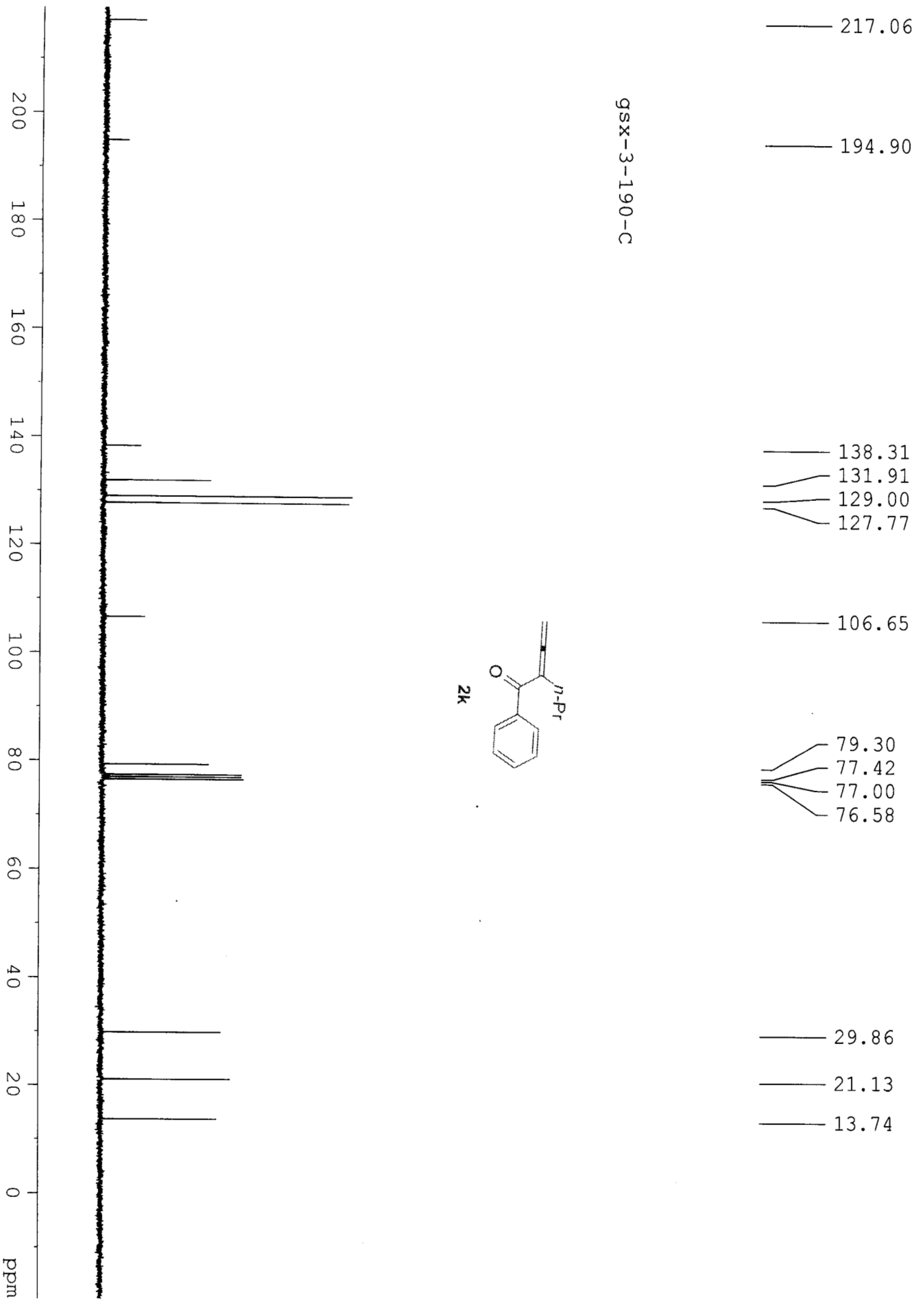
gsx-3-190-C
2k
O
n-Pr
217.06
194.90
138.31
131.91
129.00
127.77
106.65
79.30
77.42
77.00
76.58
29.86
21.13
13.74
200
180
160
140
120
100
80
60
40
20
0
ppm

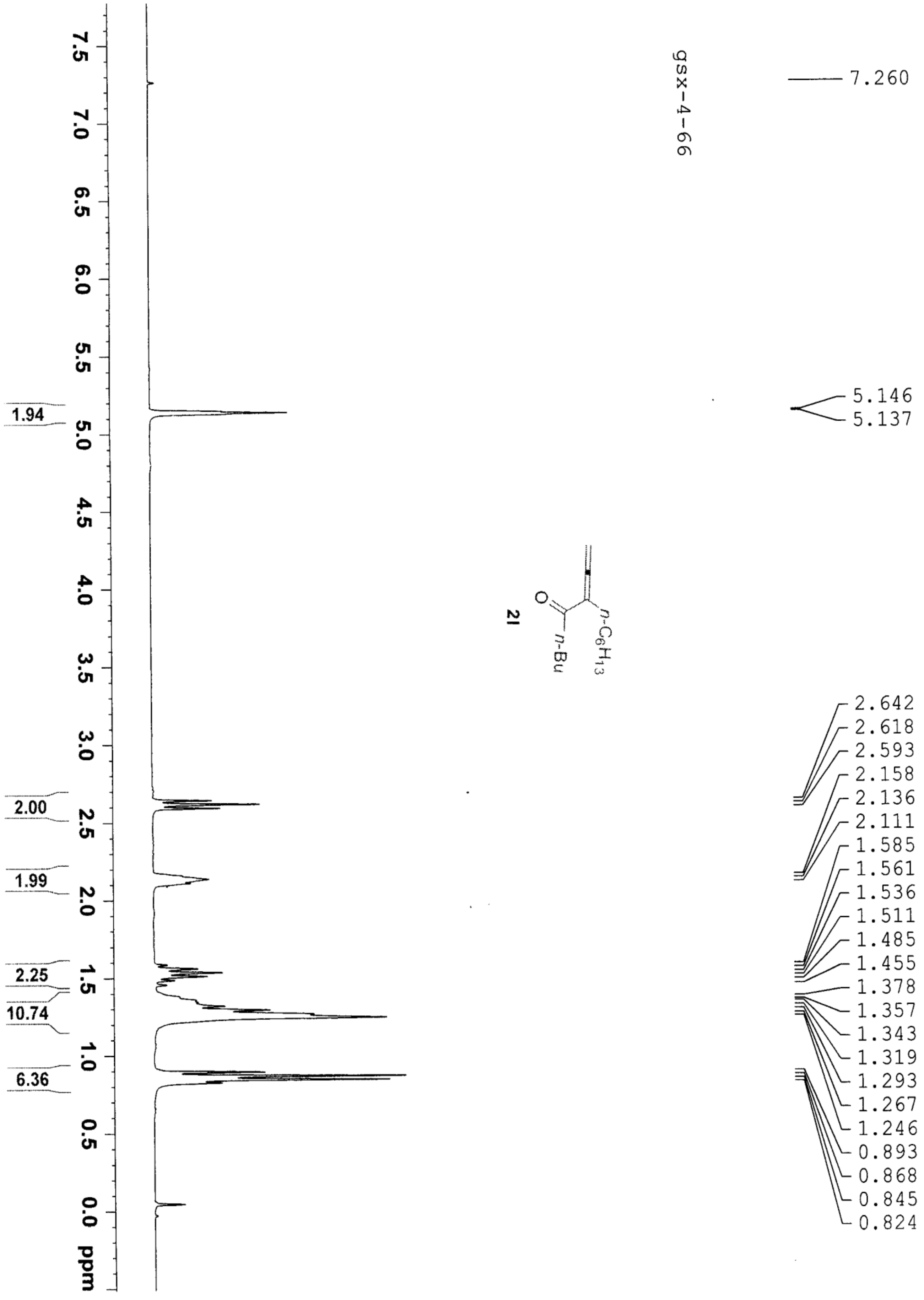
gsx-4-66
7.260
5.146
5.137
2.642
2.618
2.593
2.158
2.136
2.111
1.585
1.561
1.536
1.511
1.485
1.455
1.378
1.357
1.343
1.319
1.293
1.267
1.246
0.893
0.868
0.845
0.824
n-C6H13
n-Bu
O
2l
1.94
2.00
1.99
2.25
10.74
6.36
7.5
7.0
6.5
6.0
5.5
5.0
4.5
4.0
3.5
3.0
2.5
2.0
1.5
1.0
0.5
0.0
ppm

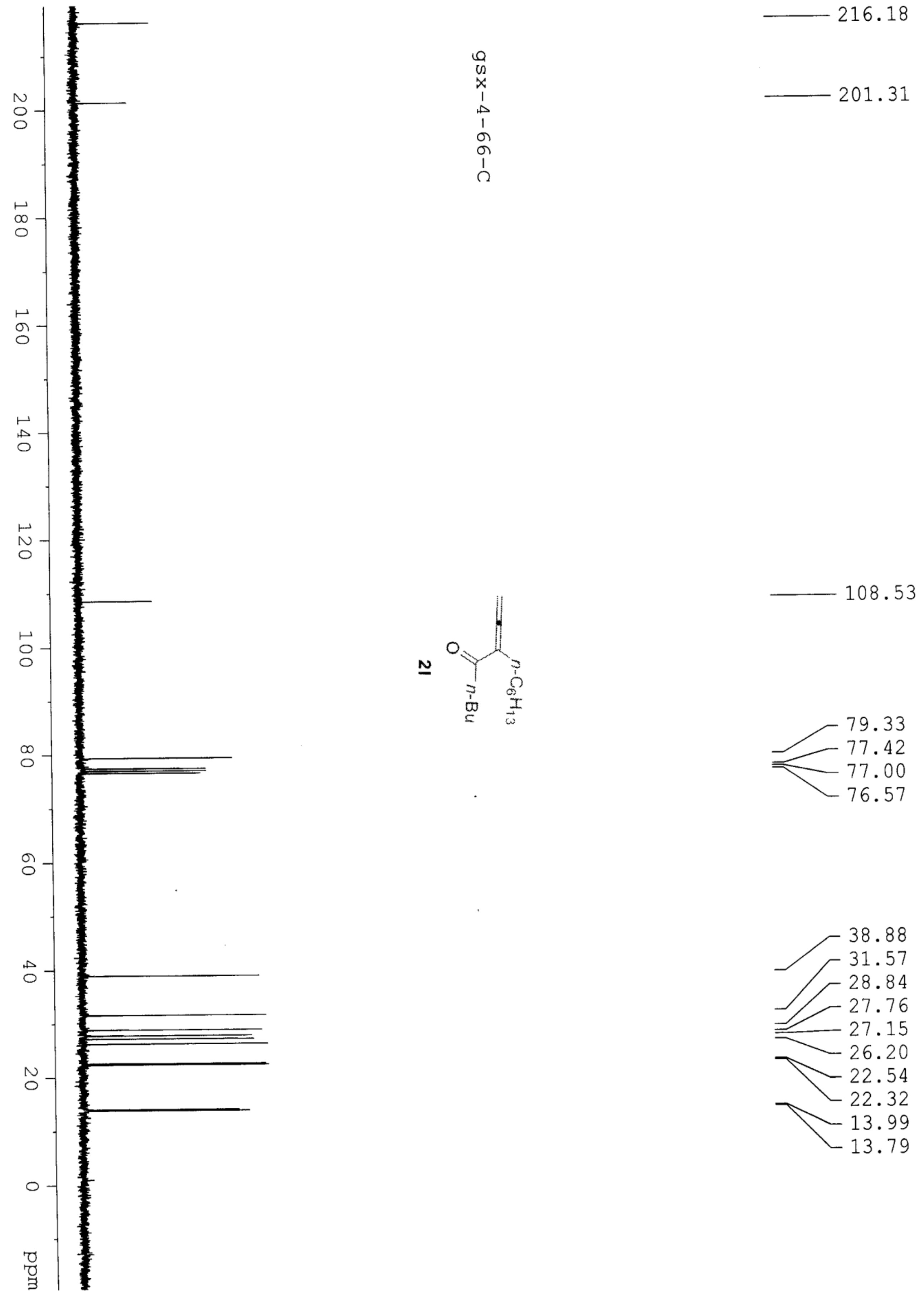
gsx-4-66-C
2l
n-C6H13
n-Bu
O
216.18
201.31
108.53
79.33
77.42
77.00
76.57
38.88
31.57
28.84
27.76
27.15
26.20
22.54
22.32
13.99
13.79
200
180
160
140
120
100
80
60
40
20
0
ppm

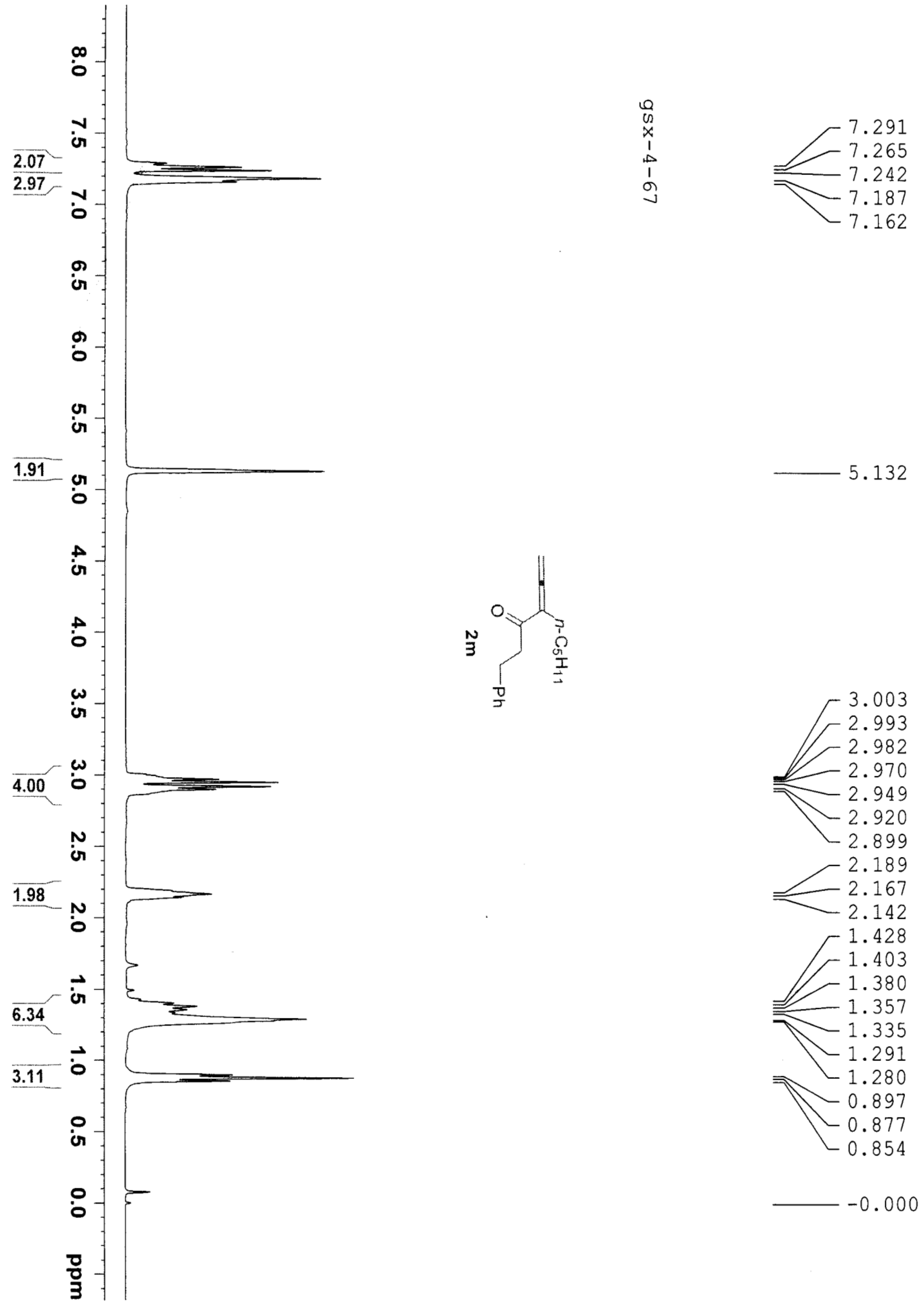
gsx-4-67
7.291
7.265
7.242
7.187
7.162
5.132
3.003
2.993
2.982
2.970
2.949
2.920
2.899
2.189
2.167
2.142
1.428
1.403
1.380
1.357
1.335
1.291
1.280
0.897
0.877
0.854
-0.000
2.07
2.97
1.91
4.00
1.98
6.34
3.11
8.0
7.5
7.0
6.5
6.0
5.5
5.0
4.5
4.0
3.5
3.0
2.5
2.0
1.5
1.0
0.5
0.0
ppm
O
n-C5H11
Ph
2m

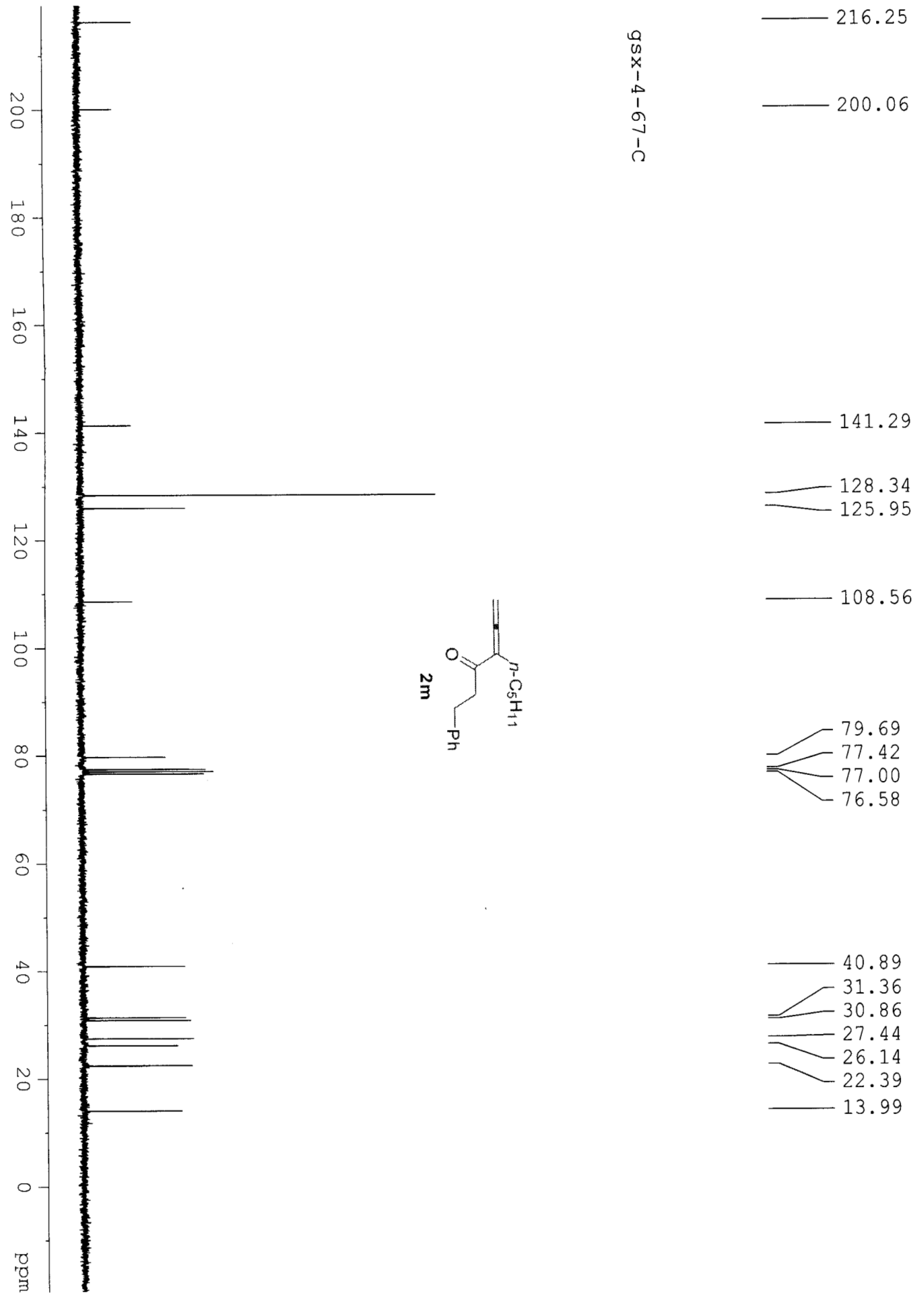
gsx-4-67-C
216.25
200.06
141.29
128.34
125.95
108.56
79.69
77.42
77.00
76.58
40.89
31.36
30.86
27.44
26.14
22.39
13.99
O
n-C5H11
Ph
2m
200
180
160
140
120
100
80
60
40
20
0
ppm
